# Genomic reconstruction of short-chain fatty acid production by the human gut microbiota

**DOI:** 10.3389/fmolb.2022.949563

**Published:** 2022-08-11

**Authors:** Maria S. Frolova, Inna A. Suvorova, Stanislav N. Iablokov, Sergei N. Petrov, Dmitry A. Rodionov

**Affiliations:** ^1^ Institute of Cell Biophysics, Russian Academy of Sciences, Pushchino, Russia; ^2^ A.A. Kharkevich Institute for Information Transmission Problems, Russian Academy of Sciences, Moscow, Russia; ^3^ Vavilov Institute of General Genetics, Russian Academy of Sciences, Moscow, Russia; ^4^ Sanford Burnham Prebys Medical Discovery Institute, La Jolla, CA, United States

**Keywords:** gut microbiome, metabolic phenotype, metagenomic, metabolic pathway, butyrate synthesis, propionate

## Abstract

Short-chain fatty acids (SCFAs) including acetate, formate, propionate, and butyrate are the end products of dietary fiber and host glycan fermentation by the human gut microbiota (HGM). SCFAs produced in the column are of utmost importance for host physiology and health. Butyrate and propionate improve gut health and play a key role in the neuroendocrine and immune systems. Prediction of HGM metabolic potential is important for understanding the influence of diet and HGM-produced metabolites on human health. We conducted a detailed metabolic reconstruction of pathways for the synthesis of SCFAs and L- and D-lactate, as additional fermentation products, in a reference set of 2,856 bacterial genomes representing strains of >800 known HGM species. The reconstructed butyrate and propionate pathways included four and three pathway variants, respectively, that start from different metabolic precursors. Altogether, we identified 48 metabolic enzymes, including five alternative enzymes in propionate pathways, and propagated their occurrences across all studied genomes. We established genomic signatures for reconstructed pathways and classified genomes according to their simplified binary phenotypes encoding the ability (“1”) or inability (“0”) of a given organism to produce SCFAs. The resulting binary phenotypes combined into a binary phenotype matrix were used to assess the SCFA synthesis potential of HGM samples from several public metagenomic studies. We report baseline and variance for Community Phenotype Indices calculated for SCFAs production capabilities in 16S metagenomic samples of intestinal microbiota from two large national cohorts (American Gut Project, UK twins), the Hadza hunter-gatherers, and the young children cohort of infants with high-risk for type 1 diabetes. We further linked the predicted SCFA metabolic capabilities with available SCFA concentrations both for *in vivo* fecal samples and *in vitro* fermentation samples from previous studies. Finally, we analyzed differential representation of individual SCFA pathway genes across several WGS metagenomic datasets. The obtained collection of SCFA pathway genes and phenotypes enables the predictive metabolic phenotype profiling of HGM datasets and enhances the *in silico* methodology to study cross-feeding interactions in the gut microbiomes.

## 1 Introduction

Short-chain fatty acids (SCFAs) are the end products of the anaerobic fermentation of non-digestible dietary fiber by the human gut microbiota (HGM). Acetate (C_2_), propionate (C_3_), butyrate (C_4_), and formate (C_1_) are major SCFAs that are produced by HGM and absorbed in the colon (Morrison and Preston, 2016). Two isoforms of lactate (D-lactate and L-lactate), although are not SCFAs, are also produced by some HGM members, such as lactic acid bacteria, bifidobacteria and others. Due to their large repertoires of carbohydrate-active enzymes, HGM bacteria are capable to degrade dietary undigested polysaccharides (i.e., resistant starch, dietary fiber) and host-produced mucus glycans and produce SCFAs and lactate as fermentation products (Briggs et al., 2021). These metabolites are subject to cross-feeding between HGM members and finally absorbed in the colon or eliminated with feces (Ríos-Covián et al., 2016). Upon absorption by various SCFA transporters expressed at different levels in the small intestine and the column (Ganapathy et al., 2013), HGM-produced SCFAs modulate key functions of the host including energy homeostasis, nutrient processing, cell proliferation, inflammatory response and immune system development (Martin-Gallausiaux et al., 2021). In particular, butyrate serves as the main energy substrate for colonocytes (Roediger, 1980). Experiments with germ free mice that lack their intestinal microbiomes demonstrate reduced colon cell proliferation, which is restored by the addition of butyrate *ex vivo* (Park et al., 2016). Some of the intestinally absorbed SCFAs also appear in the bloodstream and metabolized in the liver, at that propionate and butyrate exert larger positive biological effects than acetate. Importantly, SCFAs are involved in lipid and glucose metabolism, regulate appetite through gut hormone secretion and protect our bodies against the development of various diseases including diet-induced obesity and type 2 diabetes (Sanna et al., 2019), colorectal cancer (Louis et al., 2014), kidney disease (Gao et al., 2021), inflammatory bowel diseases (IBD) and irritable bowel syndrome (Vich Vila et al., 2018; Sun et al., 2019; Venegas et al., 2019) and nervous system disorders (Mirzaei et al., 2021).

Acetate is the most abundant SCFA produced by a large number of HGM species (Rowland et al., 2018). Acetate is directly synthesized from Acetyl-CoA (a major metabolite derived from catabolism of carbohydrates, fatty acids and amino acids) *via* an ATP-generating pathway involving phosphotransacetylase and acetate kinase, which is present in diverse bacterial species. Butyrate and propionate are usually produced in three to ten times fewer amounts than acetate by specific taxonomic groups of HGM bacteria *via* multiple alternative pathways during carbohydrate and/or amino acid fermentation (Louis and Flint, 2017). Formate is another by-product of anaerobic fermentation in many HGM species encoding pyruvate formate lyase enzyme (Rowland et al., 2018), however it is almost completely utilized by other HGM members *via* the Wood-Ljungdahl pathway resulting in the production of additional acetate (Laverde Gomez et al., 2019). Other intermediate fermentation products including lactate and succinate produced by many HGM species also do not accumulate to high levels in the colon since they are extensively involved in bacterial cross-feeding, serving as substrates for butyrate and propionate producers (Ríos-Covián et al., 2016). For example, *in vitro* co-culture studies demonstrated that acetate and lactate produced during fermentation of poly- and oligosaccharides by *Bifidobacterium* adolescentis support the growth of butyrate-producing strains of Anaerostipes, *Roseburia* and Eubacterium species with progressive increase in butyrate formation (Belenguer et al., 2006).

Diverse and important impacts of SCFAs on host physiology and health inspired a bulk of epidemiologic research and dietary intervention studies of healthy populations that measure SCFAs produced by the gut microbiota and link their metabolic profiles to health condition or diet [reviewed in (Ríos-Covián et al., 2016)]. In particular, *in vivo* dietary intervention studies allow to identify variable effects of dietary fibers on stimulation of production of SCFAs, especially butyrate, and link these effects to HGM composition (Venkataraman et al., 2016; Baxter et al., 2019; Deehan et al., 2020). On another hand, experiments on *in vitro* bacterial fermentation of HGM inoculum allow to study the effect of individual dietary compounds on metabolic SCFA profiles and taxonomic composition under simultaneous control of a few key parameters of anaerobic fermentation experiments such as medium composition, temperature, fiber particle size and glycosidic bond configuration, speed of fermentation (Reichardt et al., 2014; Harris et al., 2017; Fehlbaum et al., 2018; Chen M. et al., 2020; Miclotte et al., 2020; Thakkar et al., 2020; Yao et al., 2020; Teichmann and Cockburn, 2021). Extensive literature searches to identify *in vitro* batch fermentation studies of impact of fermentable carbohydrates on SCFA production, as recently reviewed by Harris et al. (2020), allowed the authors to classify non-digestible carbohydrate substrates according to their effect on promoting production of individual SCFA.

Butyrate-producing bacteria have been extensively studied largely by cultural studies, however their *in vitro* growth is a challenging task requiring both anaerobic conditions and specific nutrients. Majority of known butyrate producers belong to the Ruminococcaceae, Lachnospiraceae, Clostridiaceae, and Peptostreptococcaceae families within the Firmicutes phylum. In particular, *Faecalibacterium prausnitzii*, *Eubacterium rectale* and *Roseburia* spp. Are the most abundant HGM species that produce butyrate from acetate using the acetyl-CoA pathway of butyrate fermentation (Louis and Flint, 2017). Three other alternative butyrate production pathways that start from amino acid substrates (glutamate or lysine) or succinate are much less abundant among HGM Firmicutes and also very rare in other bacterial phyla (Vital et al., 2014). In contrast to butyrate, two other SCFAs acetate and propionate are mainly produced by major dietary fiber-degrading HGM species from the Bacteroidetes phylum, as well as by mucin-degrading bacteria from the Verrucomicrobia phylum such as *Akkermansia muciniphila* (Louis and Flint, 2017). Propionate is produced by HGM bacteria using three alternative metabolic pathways that start from succinate, lactate, or propanediol (Reichardt et al., 2014). The latter intermediate is formed in some HGM bacteria as a product of catabolism of the deoxy sugars rhamnose and fucose and can participate in cross-feeding between deoxy sugar degraders (e.g., Enterobacteria and *Bacteroides* species) and propionate producers encoding the propanediol pathway (such as *Roseburia* and *Blautia* species).

Our recent study of vitamin biosynthesis pathways has established a new approach for predictive functional characterization of HGM microbial communities from the 16S rRNA gene amplicon sequencing data using metabolic reconstructions in reference HGM genomes and the concept of binary metabolic phenotype that reflects the presence or absence of functional pathway variant linked to production (or utilization) of a specific metabolite (Rodionov et al., 2019). For metabolic reconstruction of target metabolic pathways we previously developed and used the subsystem-based approach implemented in SEED genomic analysis platform (Overbeek et al., 2005, 2014) that combines genomic and functional context analysis with comparative analysis of enzyme and pathway variants within the subsystem populated with genomes and functionally annotated genes (Leyn et al., 2017). This approach and the obtained genomic collection of reconstructed pathways and assigned phenotypes for vitamin biosynthesis were further used by us to assess biosynthetic potential for B-vitamins over a large collection of 16S HGM samples from generally healthy cohorts (Rodionov et al., 2019). The obtained distributions of Community Phenotype Indices (CPI) for vitamin prototrophy allow us to formulate vitamin sharing hypothesis, which was further validated in follow-up experimental study of the effect of vitamin supplementation on HGM composition and CPI values in gnotobiotic mice model (Sharma et al., 2019). The metabolic phenotype profiling approach was used in multiple subsequent studies to assess functional potential of HGM samples from fecal samples from various cohorts of participants (Delannoy-Bruno et al., 2021; Jiang et al., 2021), or *in vitro* fermentation studies to measure the influence of prebiotics and other compounds on HGM samples (Peterson et al., 2019a, 2019b, 2021; Elmén et al., 2020). In our recent paper we used machine learning techniques to show that the amount of butyrate and propionate producers represents an interpretable feature, which is stable across different datasets and, hence, can be used for distinguishing between healthy and unhealthy (Crown’s disease) groups (Iablokov S. N. et al., 2021). It was observed that higher amounts of butyrate producers are associated with healthy patients, with the opposite being true for the propionate producing bacteria. Thus, the indirect determination of SCFAs using taxonomic profiling has already been encountered in earlier studies.

In the current study, we established the bioinformatics approach for assessing the metabolic potential for SCFA synthesis in HGM metagenomic samples in both 16S rRNA gene sequencing format and whole genome sequencing (WGS) datasets. For this purpose, a detailed analysis and reconstruction of the SCFA synthesis pathways was carried out in the reference set of HGM microbial genomes. Genomic signatures were established for many variants of enzymatic pathways and bacterial species were classified according to their ability (or inability) to produce SCFAs using a simplified binary matrix of phenotypes. We also reconstructed reference metabolic pathways for production of D- and L-lactate as an additional end product of microbial fermentation. The obtained matrix of binary phenotype values for SCFAs and lactate was used for the predictive phenotype profiling of the human gut microbiome with the publicly available 16S and WGS metagenomic datasets. As result, we compared the distribution of distinct pathway variants in HGM metagenomics datasets, compared the predicted metabolic potentials with metabolomics data for butyrate and propionate and demostrated the age-dependent increase of butyrate producers in childrens microbiomes. The observed correlations and dependencies are expected to guide the development of personalized nutritional supplements and health food products.

## 2 Materials and methods

### 2.1 Selection of reference HGM genomes

The initial reference set of 2,228 human gut bacterial genomes from our previous study of vitamin metabolism (Rodionov et al., 2019) was expanded by about 450 genomes of intestinal bacteria from the PATRIC database (Wattam et al., 2017), and also included ∼150 newly sequenced HGM isolates (Gehrig et al., 2019; Chen R. Y. et al., 2020; Feng et al., 2020). The obtained genomic collection included 2,856 genomes representing eleven phyla, 43 orders, 104 families, 296 genera, 823 distinct species and 278 genomes without taxonomically defined species names (Supplementary Table S1). The largest number of selected reference genomes belong to the Firmicutes (1,331 genomes), Proteobacteria (636 genomes), Actinobacteria (504 genomes) and Bacteroidetes (303 genomes) phyla. The Fusobacteria and Tenericutes phyla are represented by 44 and 25 genomes, respectively. The remaining five phyla (Verrucomicrobia, Synergistetes, Spirochaetes, Lentisphaerae, and Planctomycetes) contain from one to six reference genomes. Phylogenetic tree of representative genomes for each genus was constructed using RaxML using concatenated alignment of ribosomal proteins as previously described (Rodionov et al., 2019) and further visualized with predicted metabolic phenotypes using iTOL (Letunic and Bork, 2021).

### 2.2 Metabolic reconstruction in reference HGM genomes

We used a subsystem-based comparative genomic approach implemented in the SEED genomic platform/database (Overbeek et al., 2005, 2014) for genomic reconstruction of metabolic pathways for production of short-chain fatty acids (formate, acetate, propionate, butyrate) and lactate in the reference set of HGM bacterial genomes (Supplementary Figure S1). For metabolic reconstruction of SCFA and lactate production pathways we collected the existing knowledge on participating biochemical reactions and Enzyme Commission (EC) numbers from literature (Vital et al., 2014; Gonzalez-Garcia et al., 2017; Louis and Flint, 2017), as well as from the Kyoto Encyclopedia of Genes and Genomes (KEGG) (Kanehisa et al., 2014) and MetaCyc (Caspi et al., 2016) databases. The identified metabolic enzymes were mapped to the analyzed reference set of bacterial genomes and are captured in the corresponding subsystems in the web-based mcSEED (microbial community SEED) environment, a local copy of the publicly available SEED database (Overbeek et al., 2005, 2014). The mcSEED subsystems were further extensively curated and expanded to identify and functionally annotate: 1) distantly related homologs of known SCFA pathway enzymes, and 2) novel enzymes that substitute missing functional roles in subsets of genomes with incomplete SCFA production pathways. Previously uncharacterized metabolic enzymes including candidates for non-orthologous gene displacements in the analyzed SCFA production pathways were initially described by analyzing their protein families in the Pfam database (El-Gebali et al., 2019), and by BLASTP similarity searches against UniProt database (Bateman, 2019) and literature using PaperBLAST (Price and Arkin, 2017). In addition to homology-based methods, we used genomic context techniques for functional gene annotation, including: 1) clustering of functionally related genes on the chromosome, and 2) patterns of occurrence of genes from the same metabolic pathway across the analyzed genomes.

### 2.3 Phenotype rules and binary phenotype matrix

**FIGURE 1:**
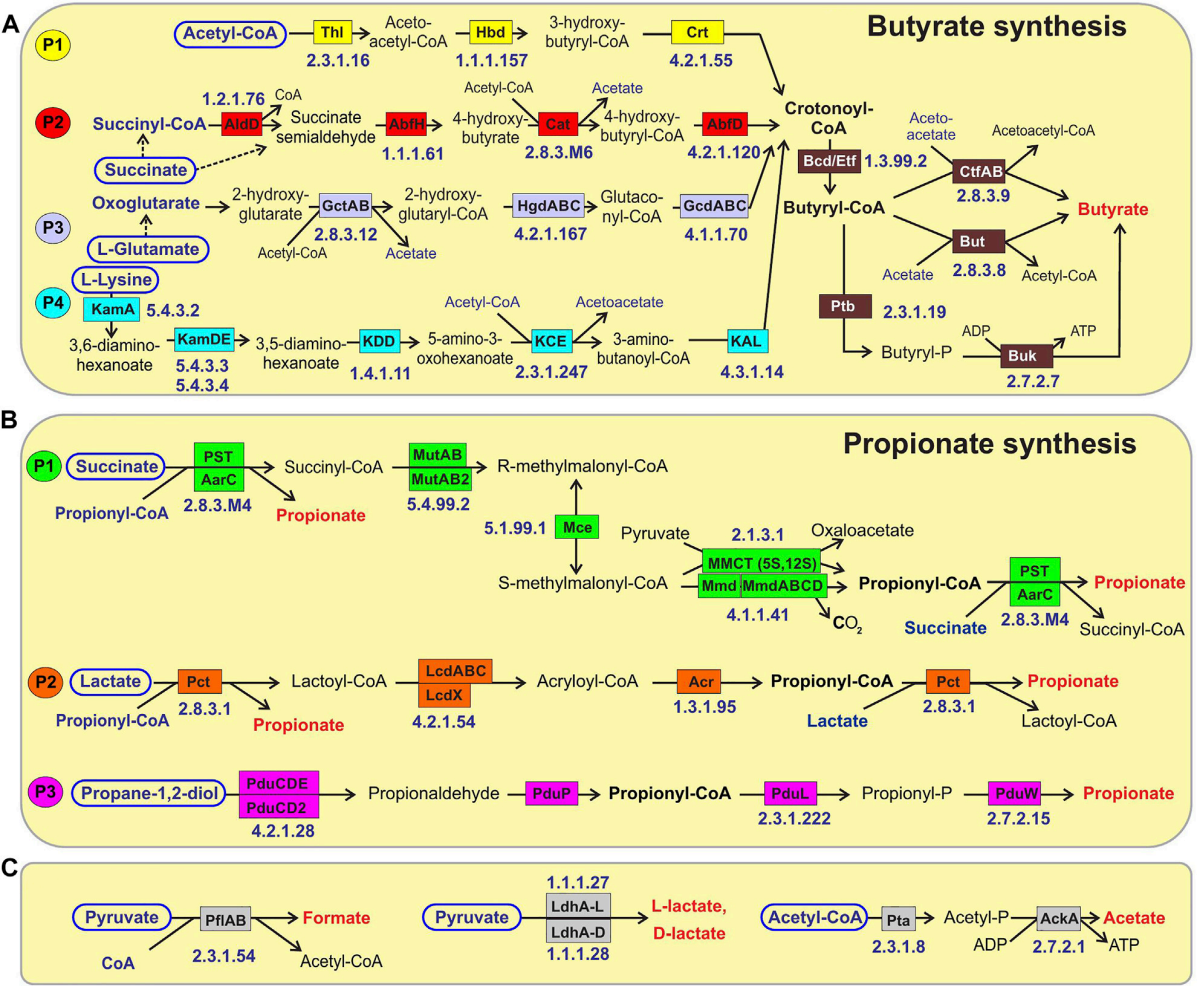
Reconstructed metabolic pathways of SCFA synthesis in reference HGM genomes. **(A)** Butyrate synthesis, **(B)** Propionate synthesis, **(C)** Acetate, Formate and Lactate synthesis. Enzymes are shown by colored boxes with indicated Enzyme Commission (EC) numbers with detailed functional roles described in Supplementary Table S1. Alternative biochemical pathways for butyrate and propionate synthesis are highlighted by different colors. Shared biochemical routes for conversion of crotonoyl-CoA to butyrate are in dark brown boxes. Central carbon metabolism metabolites and amino acids serving as substrates for acid fermentation pathways are circled; final fermentation products are in red..

The reconstructed SCFA and lactate production pathways (Figure 1) were further analyzed across 2,856 reference genomes to establish specific phenotype rules bridging the patterns of functionally annotated genes with corresponding binary metabolic phenotypes. For each analyzed metabolic pathway including alternative biochemical routes for propionate and butyrate synthesis we deduced generalized genomic signatures (subsets of metabolic enzymes) and then assigned individual pathway variant (such as P1, P2, etc.) or a combination of variants (e.g., P12) if a genome encodes two or more pathway variants for the same SCFA product (Table 1, Supplementary Table S1). For further quantitative analysis, we translated the identified pathway variants to binary phenotypes corresponding to SCFA producers (“1”) or non-producers (“0”). The obtained binary values are summarized in the form of a Binary Phenotype Matrix (BPM) capturing inferred 1/0 values for six fermentation products (butyrate, propionate, acetate, formate, L-lactate and D-lactate) derived from reconstructed metabolic subsystems across all reference genomes. This reference BPM was further used to assess the metabolic potential for SCFA synthesis in HGM metagenomic samples.

**TABLE 1:** The distribution of SCFA production pathways in the HGM reference genomes.

Fermentation product	Pathway variants^a^	# Genomes	# Species	Top taxonomic groups
Butyrate	P1	186	84	Clostridiaceae*,* Eubacteriaceae*,* Lachnospiraceae*,* Streptococcaceae
	P1+P2	41	17	*Clostridiales*
	P1+P3	35	22	*Clostridiales,* Acidaminococcaceae*,* Peptoniphilaceae
	P1+P4	28	19	*Clostridiales, Butyricimonas, Odoribacter*
	P1+P2+P3	8	2	*Clostridium, Lachnoclostridium*
	P1+P2+P4	16	10	*Porphyromonas, Clostridiales*
	P2+P4	2	1	*Paraclostridium*
	P1+P3+P4	28	5	*Fusobacterium*
	P1+P2+P3+P4	8	2	*Fusobacterium*
	P2	5	5	*Lachnoclostridium, Porphyromonas, Tannerella*
	P4	2	2	*Alistipes, Micromonospora*
Total	P1 or P2 or P3 or P4	359	164	
Propionate	P1	447	131	*Bacteroidetes, Firmicutes, Akkermansia, Enterobacteria, Actinobacteria (Propionibacteria/Corynebacteria)*
	P1+P2	6	3	Peptostreptococcaceae
	P1+P3	96	30	*Enterobacteria,* Veillonellaceae
	P1+P2+P3	2	1	*Intestinimonas*
	P2	64	20	Clostridiaceae*,* Lachnospiraceae*,* Peptostreptococcaceae
	P2+P3	18	9	Clostridiaceae*,* Eubacteriaceae*,* Peptostreptococcaceae
	P3	193	63	Diverse *Clostridia, Peptoniphilus, Veillonella, Lactobacillus, Enterococcus, Listeria, Enterobacteria, Fusobacterium*
Total	P1 or P2 or P3	826	247	
Acetate	A	2,481	686	All taxa
Formate	F	2041	534	*Actinobacteria, Bacteroidales, Enterobacteria, Firmicutes*
Lactate	L	1,153	280	*Actinobacteria, Firmicutes*
	D	632	190	*Bacteroidales, Gammaproteobacteria*
	L + D	655	200	*Fusobacterium, Firmicutes, Gammaproteobacteria*
Total	L or D	2,438	654	

a Butyrate producing pathways: P1, Acetyl-CoA; P2, succinate; P3, glutamate; P4, lysine; Propionate producing pathways: P1, succinate; P2, lactate; P3, propanediol; Lactate producing pathways: L, L-lactate; D, D-lactate.

### 2.4 Metabolic phenotype profiling of 16S microbiome datasets

For predictive profiling of SCFA production capabilities in HGM metagenomics samples, we analyzed raw 16S rRNA gene sequencing data from two large studies of broad Western populations obtained in the framework of the American Gut Project (AGP, 2868 samples) (McDonald et al., 2018) and the UK twins (UKT, 3,288 samples) study (Goodrich et al., 2016), as well as the Sweden cohort of young children (3,558 samples) from the Environmental Determinants of Diabetes in the Young (TEDDY) dataset (Stewart et al., 2018) containing longitudinal stool samples from children between 3 and 46 months of age (Stewart et al., 2018). We also analyzed the Hadza 16S metagenomics dataset representing 222 HGM samples collected from a rural community of the Hadza hunter-gatherers from Tanzania (Schnorr et al., 2014). To analyze Spearman correlation between measured SCFA concentrations and predicted production capabilities, we also analyzed 16S datasets from published *in vivo* and *in vitro* fermentation studies of HGM microbiota (Chen M. et al., 2020; Deehan et al., 2020; Elmén et al., 2020). The 16S amplicon sequencing data from all of these datasets were analyzed using the dada2 plugin in QIIME2 (Caporaso et al., 2010). Briefly, 16S sequences were quality-filtered, chimeric reads were removed, and the resulting reads were dereplicated into amplicon sequence variants (ASV) with default dada2 parameters. Taxonomic classification of the obtained ASV sequences was carried out using the multi-taxonomic assignment (MTA) approach (Iablokov S. et al., 2021) using the joined reference NCBI 16S and RDP (Cole et al., 2014) databases, with taxonomic names in RDP updated according the NCBI Taxonomy database. Finally, we renormalized the original relative abundances of ASVs by 16S rRNA gene copy numbers derived from the rrndb (version 5.6) database (Stoddard et al., 2015).

We used a development version of the Phenotype Profiler tool provided by PhenoBiome Inc (Walnut Creek, CA, United States) to assess the metabolic potential for SCFA production (see Supplementary Figure S1, part II for the workflow scheme). As previously described (Rodionov et al., 2019), we mapped ASVs to the reference organisms in BPM to assign Phenotype Indices (PI) of each ASV on the scale from 0 to 1, reflecting the probability for a given ASV to be a particular metabolic phenotype carrier. Community Phenotype Indices (CPI) were calculated for each metagenomic sample as a sum of abundance-weighted PI values, thus representing the expected fraction of community’s bacterial cells with a given metabolic capability (phenotype).

$$\text{CPI}\;\;=\sum_{i}A_{i}PI_{i}$$

where *A*_*i*_ and *PI*_*i*_ are relative abundance and phenotype index for each ASV.

A prediction error for CPI values (reflecting imprecise mapping and phenotype microheterogeneity) was calculated as:

$$\sigma =\sqrt{\sum_{i}A_{i}^{2}\left(1-PI_{i}\right)PI_{i}}$$

For ASV mapping to the HGM reference genomes, we used the threshold for nucleotide identity 90% that roughly corresponds to the taxonomic resolution on the family level. ASVs with identity to the HGM reference organisms below this threshold were discarded. According to our previous analyses, PI values were calculated by phenotype averaging across all genomes mapped with 90% threshold, resulting in PI predictions of sufficient accuracy for the majority of phenotypes, including SCFAs (Iablokov S. et al., 2021).

Metagenomic samples with less than 75% abundance coverage to the HGM reference genomes were discarded from further analysis due to higher prediction error for CPI values. The choice of this threshold is a trade-off between 1) keeping a large fraction of initial samples for better statistical significance and 2) using CPI predictions with the smallest CPI error values. For all analyzed 16S datasets except Hadza, application of 75% threshold for the abundance of “mapped” ASVs has resulted in filtering out no more than 10% of samples (see summary in Supplementary Table S4J). The Hadza dataset was characterized by the smallest mean abundance coverge by our reference HGM genomic collection (67.5%), thus resulting in only ∼1/3 of the analyzed samples retained for the phenotype profiling.

### 2.5 Functional and taxonomic profiling of WGS metagenomic samples

We analyzed shotgun metagenomic sequencing samples from two large studies: 1) the Integrative Human Microbiome Project (iHMP) that includes WGS and other multi-omics data on functional dysbiosis in the gut microbiome during IBD activity (Lloyd-Price et al., 2019); 2) and the German cohort of young children from the TEDDY dataset (Stewart et al., 2018) (1,048 samples). At first, we filtered the analyzed WGS fastq files to remove host-specific reads using Bowtie2 (Langmead and Salzberg, 2012), and the hg38 human genome assembly. We further performed quality filtering of WGS reads using the Kneaddata package (McIver et al., 2021) to enable adapter removal, trimming and filtering by quality. To obtain taxonomic profiles of WGS samples we used Kraken 2 (Wood et al., 2019) and Bracken (Lu et al., 2017) and our custom genomic database containing 2,856 reference HGM bacteria with taxonomic assignments according to the NCBI Taxonomy database. To assess the metabolic potential for SCFA production in WGS samples, we used the Phenotype Profiler tool with the relative taxonomy abundance profiles provided as an input and a taxonomy-based approach to map the respective taxonomic assignments to the reference BPM organisms (for details, see (Iablokov S. et al., 2021)). WGS entries with taxonomic similarity to the BPM worse than on family level were marked as “non-mapped” and discarded, with the respective relative abundances of “mapped” entries renormalized to sum to 1. As result, the predicted metabolic phenotype profiles included CPI values calculated for each WGS sample and each analyzed SCFA phenotype.

For gene-based functional profiling of trimmed and filtered WGS data files we implemented a pipeline including the following public domain tools: a metagenome assembly with MEGAHIT (Li et al., 2015); gene prediction with Prokka (v1.14, metagenomic mode) (Seemann, 2014); functional annotation by protein similarity search with DIAMOND (Buchfink et al., 2015); and mapping of WGS reads to the functionally annotated genes using Bowtie2 (Langmead and Salzberg, 2012). For functional annotation we used complete proteomes of 2,856 reference HGM genomes that include both functionally annotated proteins from the reconstructed metabolic pathways and representative sequences of all other proteins from these genomes. Finally, we sum up the number of mapped reads for genes with the same functional role from the reconstructed metabolic pathways using Bedtools (Patwardhan et al., 2019). At the final step, we performed gene count normalization using the trimmed mean of M-values (TMM) approach (Robinson and Oshlack, 2010) implemented in the edgeR (Robinson et al., 2010) package. For TMM-normalization, we used a core gene set that is a set of universal single-copy genes that are present in all genomes in our reference database. The gene count matrix included only genes that either belong to a set of the functionally annotated genes from the studied SCFA production pathways or genes from the core gene set. As result, the predicted functional gene profiles included TMM-normalized total abundances of genes encoding pathway-specific reactions in each SCFA production pathway variant.

### 2.6 Comparison of predicted phenotype profiles with PICRUSt2

To compare the CPI-based phenotype profiling approach with a state of the art predictive metabolic pathway abundance approach we used ASV sequences and abundance tables obtained for the AGP and UKT datasets and run PICRUSt2 with default parameters (Douglas et al., 2020). The default use case for PICRUSt2 allows one to predict: 1) abundance of KEGG ortholog (KO) families, and 2) abundance of known metabolic pathways from the MetaCyc database (Caspi et al., 2016) using KO functional annotations and the Minimal Set of Pathways (MinPath) (Ye and Doak, 2009). The predicted abundances of MetaCyc metabolic pathways for propionate and butyrate production were normalized by a number of reads in each sample. We further used the PICRUSt2 algorithm to predict abundances of binary metabolic phenotypes in the UKT and AGP datasets using the obtained BPM for SCFA production in 2,856 reference genomes. First, we mapped genomes from the PICRUSt2 reference tree to the BPM genomes using their NCBI TaxIDs and thus prepared a custom traits table for 2,607 leaves. Then, we use dthis SCFA BPM trait table with the PICRUSt2 pipeline to calculate cumulative phenotype abundances in 16S samples and finally normalize them by a number of reads.

### 2.7 Statistical analysis

The trimmed mean M (TMM) approach (Robinson and Oshlack, 2010) implemented in the edgeR package (Robinson et al., 2010) was used to normalize the number of genes for gene function prediction analysis in WGS datasets.

To detect changes of bacterial species in the butyrate-producing communities at different stages of child development in the TEDDY 16S dataset, it was performed linear discriminant analysis with effect size (LEfSe) (Segata et al., 2011). For LEfSe, the nonparametric factorial Kruskal–Wallis sum-rank test were implemented to identify taxa with significant differential abundance, the effect size was calculated by next linear discriminant analysis.

Alpha diversity (AD) and beta diversity (BD) metrics for 16S metagenomic samples were calcucated by QIIME2 (Caporaso et al., 2010) as the Faith’s phylogenetic diversity (Faith, 1992) and weighted UniFrac (Lozupone and Knight, 2005) metrics, respectively. To investigate the impact of metabolic phenotypes on BD, we used the binary SCFA production phenotypes assigned to ASVs in each sample and calculated Phenotype Beta Diversity (PBD) for the sub-communities of carriers of a particular phenotype (e.g., butyrate producers) as previously described (Iablokov S. et al., 2021). Additionally, we calculated relative PBD (rPBD) as a ratio between PBD for a given phenotype and the total BD in order to account for the possible diversity scale inheritance.

All correlation analyzes were performed using Spearman’s rank correlation by R software 4.1.2. Statistical significance was set at *p* < 0.05.

## 3 Results

### 3.1 Genomic reconstruction of SCFA synthesis pathways

We used a subsystem-based comparative genomics approach implemented in the SEED genomic database and analysis platform (Overbeek et al., 2005; Osterman et al., 2010) to reconstruct metabolic pathways for SCFA synthesis (Figure 1) in the reference collection of 2,856 bacterial genomes representing the human gut microbiome (see Supplementary Figure S1 for overall workflow scheme and Methods for details). We created three metabolic subsystem tables, corresponding to functional roles involved in the fermentation pathways of central metabolic intermediates producing butyrate, propionate and three other metabolic products, namely acetate, formate and lactate (Supplementary Table S1). Each metabolic subsystem was populated by occurrence of specific functional roles in the analyzed set of 2,856 bacterial genomes. As the result of genomic reconstruction, the three metabolic subsystems were populated by 77 functional roles including 48 biosynthetic enzymes catalyzing 38 distinct biochemical reactions (each corresponds to a unique EC number). The reconstructed metabolic pathways included several alternative pathway variants (Table 1 and Figure 1) including four pathways for synthesis of butyrate and three distinct propionate pathways, as well as alternative enzymes represented by non-orthologous gene displacements that catalyze five biochemical reactions in the propionate pathways. Additional details on the reconstructed pathways are provided below.

### 3.1.1 Butyrate synthesis pathways

The reconstructed butyrate synthesis pathways in reference HGM bacteria are represented by four pathway variants (further denoted as P1-P4) that start from acetyl-CoA (derived from pyruvate), succinyl-CoA (derived from succinate), 2-oxoglutarate (derived from L-glutamate), and L-lysine, respectively (Figure 1A). Despite different metabolic origins, all four butyrate pathways merge at a common intermediate, crotonyl-CoA, which is converted to butyryl-CoA *via* the universal reaction by the butyryl-CoA dehydrogenase electron-transferring flavoprotein complex Bcd-EtfAB (Vital et al., 2014; Louis and Flint, 2017). P1 pathway is closely related to the beta-oxidation of fatty acids; here crotonyl-CoA is synthesized in a three-step process, where two molecules of acetyl-CoA are converted into acetoacetyl-CoA *via* thiolase Thl, and it is further reduced and dehydrated by dehydrogenase Hbd and dehydratase Crt, respectively. In P2 pathway, succinyl-CoA is reduced to 4-hydroxybutyrate *via* CoA-dependent succinate-semialdehyde dehydrogenase AldD and NAD-dependent 4-hydroxybutyrate dehydrogenase AbfH, which is further transformed to crotonyl-CoA by transferase Cat and dehydratase AbfD. In P3 pathway, 2-oxoglutarate is reduced to 2-hydroxyglutarate and then to corresponding CoA-intermediate by glutaconate CoA-transferase GctAB, and further transformed into crotonyl-CoA *via* the 2-hydroxyglutaryl-CoA dehydratase HgdABC and glutaconyl-CoA decarboxylase GcdABC, respectively. P4 pathway includes transformation of L-lysine *via* the KamA and KamDE mutases into an intermediate, which is further deaminated and oxidized by dehydrogenase KDD, cleaved by KCE, and deaminated *via* lyase KAL, yielding crotonyl-CoA. The final conversion of butyryl-CoA to butyrate is carried out either directly by various butyryl-CoA transferases, such as CtfAB or But, or through butyryl-phosphate *via* transferase Ptb and kinase Buk.

Out of 2,856 studied genomes, complete pathways of butyrate synthesis were identified only in 359 genomes, with the most common P1 pathway variant present in 350 of them (Table 1). In about half of genomes with P1 pathway, it was found concurrently with the other variants of butyrate synthesis in different combinations. Only nine studied genomes encoded the P2 and/or P4 pathway variants in the absence of the P1 variant, while the P3 pathway variant always co-occurs with P1. Combinations of three or four pathway variants for butyrate synthesis in a single genome are quite rare, the most prominent example being the *Fusobacterium* species. Taxonomically, pathways of butyrate synthesis are most widely distributed among the Firmicutes (mainly from the Clostridia class), and, to a much lesser extent, among a few genera from the Bacteroidetes, Fusobacteria, and Spirochaetes phyla (Supplementary Figure S2), while are almost completely (apart from a few species) absent in the Actinobacteria and Proteobacteria phyla. These observations are in agreement with previously published data [(Vital et al., 2014; Louis and Flint, 2017) and see below].

### 3.1.2 Propionate synthesis pathways

The reconstructed propionate production pathways in reference HGM bacteria include three previously described variants (P1-P3) that make propionate from either succinate, lactate, or propanediol metabolic precursors, respectively (Figure 1B). Lactate and succinate are produced from pyruvate and oxaloacetate during catabolism of many carbohydrates and amino acids (Vital et al., 2014; Gonzalez-Garcia et al., 2017; Louis and Flint, 2017), while propanediol is a product of anaerobic catabolism of two methyl-pentoses, L-fucose and L-rhamnose (Baldomà and Aguilar, 1988). In the P1 pathway, succinate is converted to succinyl-CoA *via* either AarC or PST transferases that concurrently transform propionyl-CoA to propionate. Succinyl-CoA is converted to propionyl-CoA in three further steps using vitamin B12-dependent methylmalonyl-CoA mutase MutAB, epimerase Mce, and methylmalonyl-CoA decarboxylase (a single-subunit enzyme Mmd or multisubunit complex MmdABCD). Alternatively, S-methylmalonyl-CoA can be converted into propionyl-CoA using pyruvate-dependent transcarboxylase MMCT that contains two subunits (5S, 12S). In P2 pathway, lactate is converted through lactoyl-CoA into acryloyl-CoA *via* CoA-transferase Pct and dehydratase LcdABC, and it is further reduced to propionyl-CoA by reductase Acr. Final release of propionate from propionyl-CoA is catalyzed by the same upstream pathway CoA-transferases both in P1 and P2 pathways (PST, AarC, Pct). P3 pathway includes propane-1,2-diol dehydration to propionaldehyde *via* B12-dependent dehydratase PduCDE, next step carried out by dehydrogenase PduP yields propionyl-CoA, which is converted to propionate by consecutive actions of transferase PduL and kinase PduW.

Among 2,856 reference genomes, complete pathways of propionate synthesis were found in 826 genomes (Table 1, Supplementary Table S1). The succinate pathway P1 is the most common variant, present in more than half of propionate producers as the only propionate synthesis pathway, and in a hundred more genomes—in combination with two other pathway variants. The P1 pathway is typical for a large number of species from the Firmicutes, *Bacteroides*, Proteobacteria and Actinobacteria phyla. The propanediol pathway P3 is the second most abundant variant found in over 300 genomes represented by ∼100 taxonomically diverse species from the Firmicutes phylum (including representatives of Bacillales, Clostridiales, Lactobacillales, Tissierellales, and Veillonellales orders), Proteobacteria (25 species from the Enterobacteriales order), and Fusobacteria. In addition, the *pdu* gene locus encoding the P3 pathway was found in a single representative from the Bacteroidetes phylum, *Bacteroides xylanolyticus*, suggesting it was a subject to a horizontal transfer from Firmicutes. The lactate (acrylate) pathway P2 was identified in 90 genomes corresponding to 30 species from the Firmicutes phylum (mostly from the Clostridia class), as well as in a single *Fusobacterium* species.

### 3.1.3 Alternative enzymes in propionate synthesis pathways

The succinate pathway P1 is the most common pathway for propionate synthesis identified in 551 HGM genomes. With the exception of Mce, all other biochemical steps in the P1 pathway were represented by two or more alternative enzymes encoded by non-orthologous genes (Figure 1B). The prevailing, multi-subunit form of methylmalonyl-CoA decarboxylase, MmdABCDE, was found in 290 genomes representing diverse bacterial phyla, while 153 genomes from the Proteobacteria phylum (mostly Enterobacteria) have a non-orthologous form of a single-subunit enzyme Mmd (Benning et al., 2000). An alternative two-subunit enzyme for the synthesis of propionyl-CoA from S-methylmalonyl-CoA, MMCT, was identified in 108 genomes from two genera of Actinobacteria (*Propionibacterium*, *Corynebacterium*). Noteworthy, all three alternative enzymes do not co-exist in any studied HGM genome. Two alternative forms of methylmalonyl-CoA mutase were identified: 1) MutAB, where both alpha and beta subunits contain fused catalytic and B12-binding domains, i.e., MutAB from *Propionibacterium shermanii* (McKie et al., 1990), and 2) MutA2-MutC, where each subunit corresponds to catalytic and B12-binding domain, respectively, as represented by a two-subunit methylmalonyl-CoA mutase from *Pyrococcus horikoshii* (Yabuta et al., 2015). We also identified an accessory GTPase protein from COG1703 family (termed MutB2) encoded in the same gene cluster with *mutA2* in many Proteobacteria and Firmicutes possessing the P1 pathway variant. The most common CoA-transferase from the P1 pathway, PST, was identified in over 400 genomes representing the Bacteroidetes, Proteobacteria and Actinobacteria phyla, while a non-orthologous alternative form of CoA-transferase, AarC, is present in 57 genomes from the Firmicutes phylum, as well as in *Akkermansia* spp. Interestingly, both PST and AarC isozymes were not found in ∼80 analyzed genomes possessing all other components of the P1 pathway including known propionate producers such as *Alistipes* spp. And *Prevotella ruminicola* (Strobel, 1992; Parker et al., 2020), suggesting the existence of yet unidentified succinate:propionyl-CoA transferase in their genomes.

The lactate pathway P2 contains two alternative forms of lactoyl-CoA dehydratases, LcdABC and LcdX. The previously characterized three-subunit enzyme LcdABC is present in 62 genomes possessing P2 pathway, whereas the novel predicted dehydratase LcdX was found in 43 genomes (Firmicutes, mostly Clostridia), including 18 genomes possessing both alternative isozymes (Supplementary Table S1). LcdX belongs to the COG1024 family from the crotonase/enoyl-CoA hydratase superfamily (cl23717), which includes 3-hydroxybutyryl-CoA dehydratase from the P1 pathways of butyrate synthesis. Analysis of genomic context suggests that *lcdX* orthologs always co-occur and co-localize with *acr* and/or *pct* genes encoding two other enzymes from the P2 pathway of propionate synthesis. Based on these observations we predicted LcdX function as lactoyl-CoA dehydratase (EC:4.2.1.54).

Finally, the propanediol pathway P3 includes two non-orthologous forms of propanediol dehydratase. The B12-dependent three-subunit enzyme PduCDE was identified in 225 out of 309 genomes containing the P3 pathway, whereas B12-independent propanediol dehydratase, named PduC2, is present in 106 genomes that represent diverse Firmicutes, but also a few *E. coli* strains, *Rhodospirillum rubrum* and *Bacteroides xylanolyticus* (pathway variant P3*). These include 28 Clostridiales genomes and *Bacillus massiliosenegalensis* posessing both alternative forms of propanediol dehydratase. The B12-independent propanediol dehydratase PduC2 is a glycyl radical enzyme that belongs to the COG 1882 (PflD) family, which includes pyruvate formate lyase 2 and B12-independent glycerol dehydratase. In all analyzed genomes, PduC2 is accompanied by the radical SAM-family proteins PduD2 that belongs to the COG1180 (PflA) family, including glycerol dehydratase activating protein, and thus is likely to function as an PduC2 activating enzyme. The predicted new propanediol dehydratase PduC2 and the PduD2 activating enzyme have been recently biochemically characterized in *Roseburia inulinivorans,* and confirmed to utilize 1,2-propanediol as a substrate in strictly anaerobic conditions (LaMattina et al., 2016).

### 3.1.4 Other SCFAs and lactate synthesis pathways

Acetate is synthesized by two consequential reactions from acetyl-CoA using acetate kinase AckA and phosphate acetyltransferase Pta (Figure 1). Formate is formed in a single-enzyme reaction from pyruvate *via* a strictly anaerobic enzyme, pyruvate formate-lyase PflB, which also requires the radical SAM-family activating enzyme PflA to regenerate the inactive PflB enzyme after each catalytic round. Both acetate and formate production pathways are widely distributed in HGM bacteria, being present in 86.8 and 71.4% of the analyzed HGM genomes, and corresponding to 686 and 530 out of 830 analyzed species, respectively.

Two isoforms of lactate are formed from pyruvate by either L-lactate dehydrogenase or D-lactate dehydrogenase (Figure 1). These fermentation pathways are also frequent among HGM bacteria: L-lactate and D-lactate pathways were identified 63.3 and 45% of the analyzed genomes, corresponding to 470 and 385 species, respectively (Supplementary Table S1). At that 200 HGM species (22.9% of analyzed genomes) possess both L- and D-lactate pathways.

### 3.2 Comparison of predicted pathways with published experimental data

To validate the predicted SCFA production capabilities in reference HGM genomes, we collected the published experimental data on SCFAs synthesis by each analyzed HGM species. As result, we found experimentally described SCFA production phenotypes for 210 out of 823 reference HGM species (Supplementary Table S2). Among 147 predicted propionate producers with experimentally data available, 125 species (85%) indeed are capable to produce propionate, while the propionate production capability has not been reported for the remaining 22 species. For butyrate production, we found experimental confirmation for 112 out of 117 predicted butyrate producers, thus giving 96% consistency between experimental and predicted phenotypes. The remaining inconsistencies may originate from a variety of factors including strain-specific phenotype variations (as exemplified by four strains of *Hungatella hathewayi*, only one of them is a predicted butyrate producer), transcriptional repression of the propionate/butyrate pathway genes in the analyzed experimental conditions, or by a significantly larger metabolic flow to generate alternative fermentation product(s). For propionate production, we also observed a few cases of inconsistencies in the opposite direction, when three HGM species that are experimentally confirmed to produce propionate, appear to lack any propionate synthesis pathway in their genomes (*Clostridium viride, Acidaminococcus intestini, Eubacterium biforme*). Finally, in the process of searching for experimental SCFA phenotype evidences in published sources, were found a few other characterized SCFA producers, including *Clostridium neopropionicum, Anaerotignum neopropionicum, Alistipes dispar* ***,*** *Alistipes communis, Anaerococcus octribumium, Butyricimonas faecalis, Clostridium phytofermentans* ***,*** *Corynebacterium avidum, Peptoniphilus ivorii, Clostridium sphenoides*. These HGM organisms have not been described in our reference genomic collection, we thus plan to add them to our collection in the future. Overall, the examined experimental data on SCFA production are in good agreement with our *in silico* reconstruction and prediction of propionate and butyrate production phenotypes.

### 3.3 Variability of SCFA binary phenotypes

To apply the reconstructed SCFA synthesis pathways in reference genomes to metagenomic HGM samples we use the concept of *binary phenotypes* introduced in our previous study of vitamin synthesis capabilities in the human gut microbiome (Rodionov et al., 2019). Binary representation of SCFA synthesis phenotypes (Supplementary Table S1) allows us to assess the phylogenetic distribution of predicted SCFAs producers across HGM reference genomes, as visualized on the phylogenetic tree constructed for representative HGM genomes for nearly 300 analyzed genera (Supplementary Figure S2). The largest analyzed phylum of Firmicutes (1,331 genomes, 134 genera) contains mostly producers capable of synthesizing all described SCFAs. The second largest phylum of Proteobacteria (636 genomes, 79 genera) contains a large number of acetate, formate and D-lactate producers, whereas L-lactate producers are much less abundant, propionate is only produced by a subset of enterobacteria, and the butyrate synthesis pathway was identified in a single genome (*Kosakonia sacchari*). Most of the 504 analyzed genomes from the Actinobacteria phylum (47 genera) are producing acetate, formate, and L-lactate, whereas propionate and D-lactate can be produced by 6 and 3 genera, respectively, and the butyrate production pathway was found only in a single genome (*Micromonospora aurantiaca*). The Fusobacteria (44 genomes, 3 genera) and Bacteroidetes (303 genomes, 24 genera) phyla contain mostly producers capable of synthesizing all described SCFAs. The Verrucomicrobia phylum as represented by six *Akkermansia* strains have biosynthetic capabilities for formate, propionate and D-lactate. Three reference genomes from the *Synergistetes* phylum are capable of formate and propionate synthesis. The Lentisphaerae phylum represented by a single HGM species (*Victivallis vadensis*) produces only acetate and formate. Both strains of *Brachyspira pilosicoli* from the Spirochaetes phylum are producers of acetate, butyrate and L-lactate. The Tenericutes phylum members (6 *Mycoplasma* and 19 *Ureaplasma* strains) are able to produce acetate and L-lactate. A single HGM strain from the Planctomycetes phylum (*Schlesneria paludicola*) can produce only L-lactate.

To assess variations of the assigned binary SCFAs synthesis phenotypes at the species and genus taxonomic levels we used two metrics: 1) *number of variable phenotypes* (NVP), and 2) *overall phenotype variability score* (OPVS) calculated as a sum of variances for each SCFA phenotype (Supplementary Table S3). The highest individual phenotype variability score 0.5 corresponds to a case when a taxonomic group is represented by an equal number of SCFA producers and non-producers, thus the cumulative OPVS metric ranges between 0 and 3 for six analyzed SCFA phenotypes. Overall, 330 out of 823 analyzed HGM species are represented by two or more strains, including 39 species with 10 or more strains. Of those, 63 species (∼7.6%) have at least one variable SCFA phenotype including 14 species characterized by two variable phenotypes (NVP = 2). These include two closely-related species of Clostridia, *C. bolteae* (7 strains) and *C. clostridioforme* (9 strains) possessing highly variable butyrate and propionate production phenotypes. Other phylogenetically diverse HGM species including *Acinetobacter baumannii* (21 strains), *Enterococcus faecalis* (62 strains), *Fusobacterium nucleatum* (21 strains) and *Fusobacterium periodonticum* (6 strains) demonstrated high OPVS values due to variability of the acetate, L-, D-lactate and propionate production phenotypes. Similar metrics were used to assess SCFA phenotype variations at the genus level. Out of 296 analyzed HGM genera, 163 were represented by more than one genome, and 102 of them (∼34%) demonstrated various degrees of phenotype variability. Majority of genera with the highest variability of SCFA binary metabolic phenotypes (NVP >3; OPVS >0.95) belong to the Clostridia class from the Firmicutes phylum (Table 2). In contrast, other HGM phyla including Bacteroidetes, Proteobacteira and Actinobacteria demonstrate relatively low levels of variations of SCFA production phenotypes. For instance, the *Bacteroides* and *Prevotella* genera from the Bacteroidetes phylum were characterized by 5 and 4 variable phenotypes with OPVS values 0.13 and 0.38, respectively. Finally, by comparing cumulative variability metrics between individual SCFA phenotypes across all analyzed HGM genomes, we demonstrated that the L-, D-lactate, formate and propionate production are most variable phenotypes both at the species and genus level, whereas the acetate and butyrate production phenotypes are more conserved in closely-related species and genera of HGM strains.

**TABLE 2:** Taxonomic genera with high variability of SCFA binary metabolic phenotypes.

HGM genus	# Strains	Variability metrics^a^		Family	Phylum
		NVP	OPVS		
Anaerotruncus	4	4	1.50	Ruminococcaceae	Firmicutes
*Clostridium*	53	4	0.98	Clostridiaceae	Firmicutes
Coprococcus	6	5	1.33	Lachnospiraceae	Firmicutes
Corynebacterium	18	4	1.06	Corynebacteriaceae	Actinobacteria
Desulfotomaculum	8	5	1.88	Peptococcaceae	Firmicutes
Eubacterium	14	6	1.79	Eubacteriaceae	Firmicutes
Lachnoclostridium	35	5	1.12	Lachnospiraceae	Firmicutes
Peptoniphilus	12	4	1.33	Peptoniphilaceae	Firmicutes
Pseudoflavonifractor	4	3	1.25	—	Firmicutes
Ruminiclostridium	9	5	1.33	Ruminococcaceae	Firmicutes
Ruminococcus	18	4	1.16	Ruminococcaceae	Firmicutes
Subdoligranulum	3	4	1.33	Ruminococcaceae	Firmicutes
Tannerella	2	3	1.50	Tannerellaceae	Bacteroidetes

a NVP, number of variable phenotypes; OPVS, overall phenotype variability score.

### 3.4 Prediction of SCFA production capacity for human gut communities

#### 3.4.1 Metabolic profiling of microbiomes from diverse national cohorts

**FIGURE 2:**
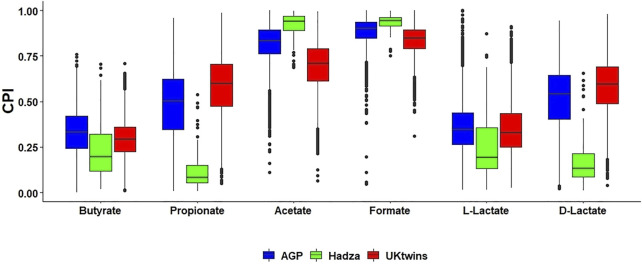
Distribution of Community Phenotype Indices (CPI) for SCFAs and lactate in HGM 16S samples from AGP, UKT and Hadza datasets. Box plots with the median values show distribution of CPI values calculated for each 16S sample. Each CPI value corresponds to the relative abundance of bacterial 16S reads possessing predicted metabolic capability to produce a SCFA.

We used the metabolic phenotype profiling approach and the obtained reference BPM (see Methods) to predict SCFA production capabilities for HGM samples from three published 16S rRNA gene sequencing datasets, namely the American Gut Project (AGP) (McDonald et al., 2018), the UK twins (UKT) (Goodrich et al., 2016) and the Hadza dataset representing the community of hunter-gatherers from Tanzania (Schnorr et al., 2014). By applying the Phenotype Profiler pipeline to ASV taxonomic profiles of each metagenomic sample, we calculated the sample-specific Community Phenotype Indices (CPI) and respective CPI prediction errors for each SCFA synthesis phenotype (Supplementary Table S4). Average CPI prediction errors, reflecting imprecise mapping of ASVs to reference genomes and phenotype microheterogeneity, are in the 0.01–0.03 range for each dataset. Each CPI value represents a predicted fraction of microorganisms that are capable for production of a corresponding SCFA. We then compared CPI distributions for each SCFA metabolic phenotype between the AGP, UKT and Hadza datasets (Figure 2). All three datasets show high average CPI values for the production of acetate and formate, while the production of butyrate and both lactate forms is characterized by significantly lower CPI values. For AGP and UKT datasets, the observed distributions share a high degree of similarity for all phenotypes, while the Hadza dataset demonstrates a different pattern. Most strikingly these differences are manifested for the propionate and D-lactate production, with the respective CPI values being significantly lower for the Hadza, as compared to the Western HGM communities. We also noticed a similar, however, less pronounced trend for a decreasing production capabilities for butyrate and L-lactate, while the acetate and formate production is slightly higher in the Hadza community.

**FIGURE 3:**
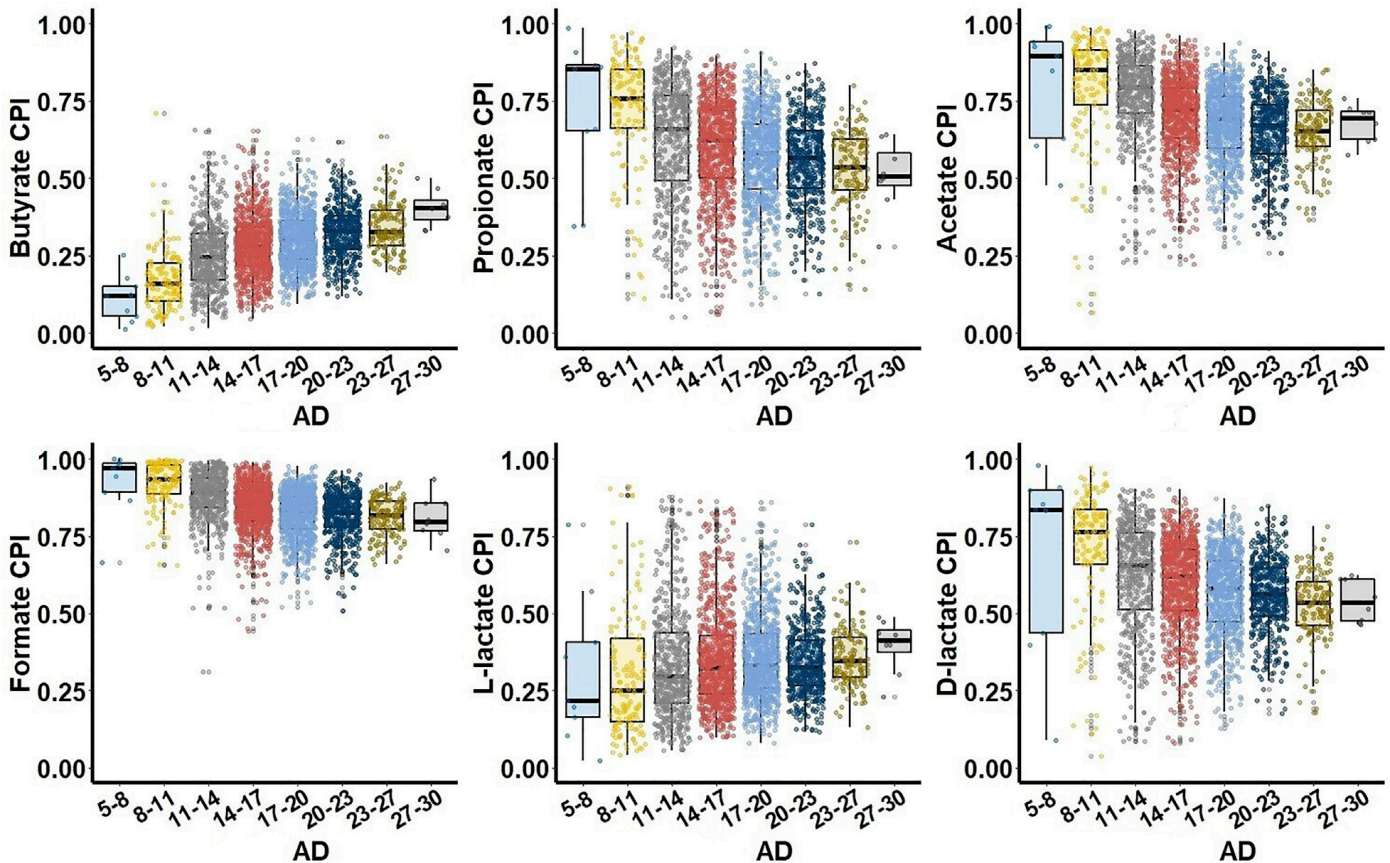
Relationship between Community Phenotype Indices (CPI) and Alpha Diversity (AD) for the UKT dataset. Samples are grouped together based on their AD values calculated using Faith phylogenetic diversity metric.

To study relationship between SCFA production phenotypes and microbial alpha diversity (AD) of the gut microbiome samples, we binned all samples into groups of similar AD and constructed CPI-vs.-AD scatterplots for each SCFA metabolic phenotype. The UKT dataset demonstrated characteristic phenotype-specific dependences between CPI and AD values (Figure 3). First, all SCFA production phenotypes can be divided into two groups of either increasing (butyrate, L-lactate) or decreasing (propionate, acetate, formate, D-lactate) median CPI values. Second, largest CPI variations for all SCFA phenotypes were observed for intermediary values of AD, while samples with the highest AD have the decreased variations in CPI values. The AGP dataset showed similar CPI-vs.-AD distributions for butyrate, acetate, formate, and lactate phenotypes, however propionate is characterized by significantly lower CPI values in samples with low AD (Supplementary Figure S3). In both AGP and UKT datasets, the samples with the highest AD values converge to similar median CPI values for each SCFA phenotype. Dietary strategy has a large impact on biodiversity of the animal gut microbial communities, when higher AD is generally associated with more stable and healthy gut (Reese and Dunn, 2018). Thus, the observed CPI values corresponding to the highest AD values can be interpreted as optimal relative abundances of SCFA producers in the healthy HGM samples.

### 3.4.2 Metabolic profiling of young children microbiomes

**FIGURE 4:**
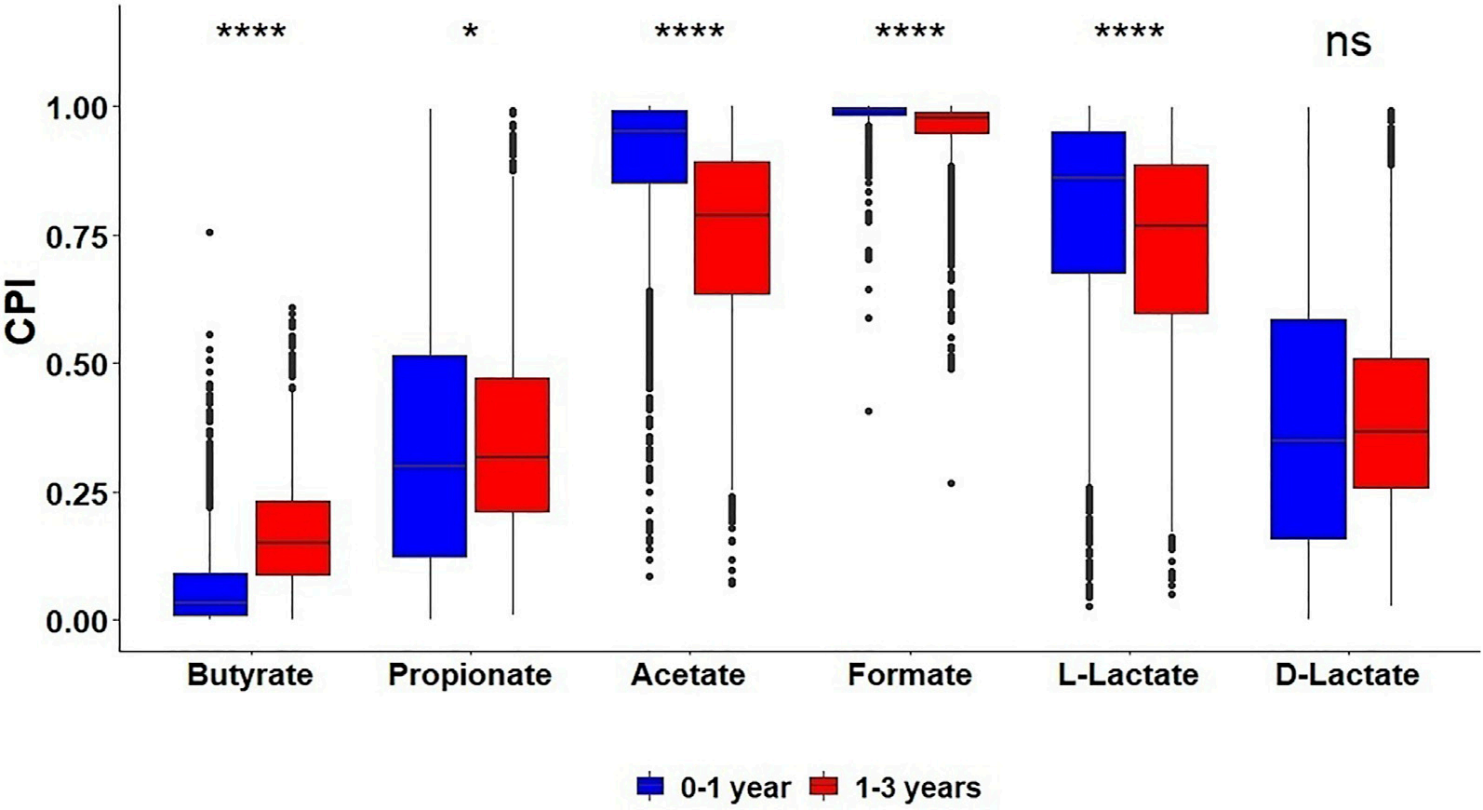
Distribution of Community Phenotype Indices (CPI) for SCFAs and lactate in HGM 16S samples from the TEDDY dataset among two age groups of children.

The gut microbiota is passed on to newborns from their mothers and develops with age (Tannock, 2021). To analyze changes in HGM metabolic capabilities during development of infant microbiota, we applied the metabolic profiling approach to 16S rRNA gene sequencing dataset for the Sweden cohort from The Environmental Determinants of Diabetes in the Young (TEDDY) study (Stewart et al., 2018). The obtained CPI values for SCFA production in 3,558 stool samples were analyzed in the context of available metadata: 1) Type 1 diabetes (T1D) status and 2) child age (between 2 months and 5 years old) (Supplementary Table S4D). First, we compared metabolic potential for SCFA production in healthy subjects and T1D patients, and found no significant differences in the corresponding distributions of CPI values between these groups for all considered metabolic phenotypes. This coincided with the previously obtained microflora-associated characteristics in the diabetes and control children from Sweden by determining their fecal concentrations of SCFAs, when no statistically significant differences were reported (Samuelsson and Ludvigsson, 2004). We further compared the SCFA production potential of these samples grouped by the children age (Figure 4). The fraction of butyrate producers, as reflected by corresponding CPI values, was significantly larger for 1–3 years old children compared to infants under 1 year old, while the sub-communities of acetate and L-lactate producers were highly reduced in older children.

**FIGURE 5:**
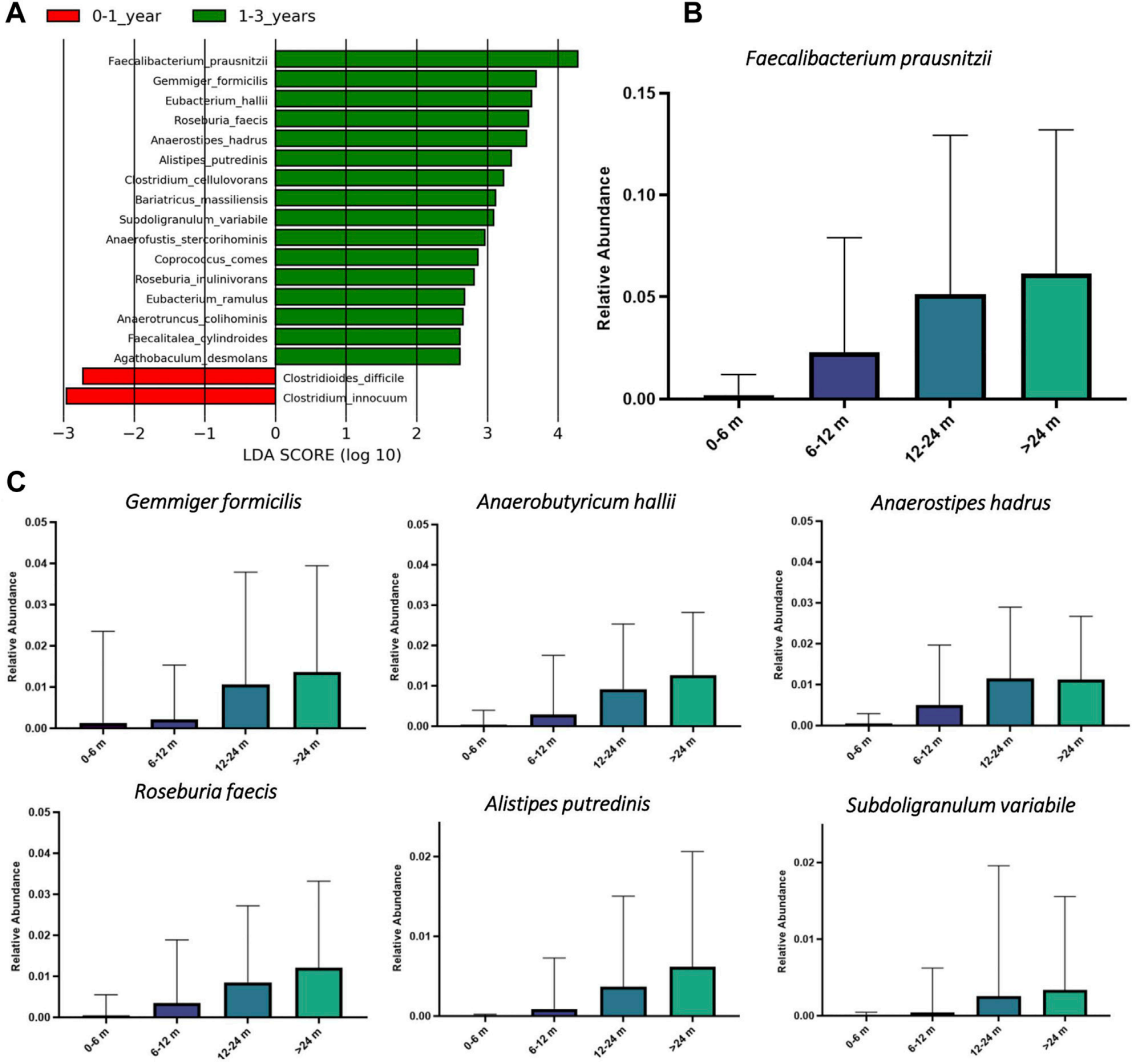
**Li**near discriminant analysis with effect size (LEfSe) for butyrate producers in HGM samples from young children of different age groups in the TEDDY study. **(A)** The LEfSe analysis was performed on taxonomic abundances of Amplicon Sequence Variants (ASVs) representing predicted butyrate producers in each sample. LDA score plot includes top taxonomic species corresponding to the most discriminative butyrate producers between two age groups of children. **(B)** and **(C)** Boxplots of relative abundances of the most dominant butyrate producing species in HGM samples from children in different age groups.

To investigate taxonomic biomarkers responsible for the observed quantitative shifts in the butyrate-producing communities, we performed linear discriminant analysis with effect size (LEfSe) (Segata et al., 2011) at different stages of child development in the TEDDY 16S dataset. LEfSe was performed after filtration of the 16S metagenomic dataset to retain only ASVs assigned to the butyrate producing species. This analysis identified 16 discriminative species (LDA score >2.8) between two age subcohorts of children (Figure 5A). These include *F. prausnitzii*, a major butyrate producing bacterium in HGM (Lopez-Siles et al., 2017), as well as other butyrate producers, namely *Gemminger formicilis*, *Anaerobutyricum hallii*, *Anaerostipes hadrus*, *Roseburia faecis*, *Alistipes putredinis* and *Subdoligranulum variabile*, that are present in a subset of samples with relative abundance >1%. We further compared relative abundances of these butyrate-producing species in children of different age groups (Figures 5B,C). Each of these identified species are either absent or significantly underrepresented in the subcohort of 0–6 month old children. In contrast, these species demonstrated gradual increase in their relative abundances after 6 months of age reaching maximal values in the 24 months and older group.

Finally, we compared microbial composition of samples within and between groups of samples from the TEDDY dataset with available metadata (age, diagnosis). In order to measure the overall scale of the observed differences, we performed beta diversity (BD) analysis and considered the average pairwise distances between samples for each comparison scenario. We found that for both intergroup and intragroup comparisons their respective BD values were of the same order of magnitude (see Supplementary Table S5), suggesting the absence of high-level taxonomic biomarkers that can distinguish the analyzed groups of samples. We further restricted the BD analysis to the sub-communities of SCFA producers and calculated Phenotype Beta Diversity (PBD) relative PBD (rPBD) and for each SCFA phenotype. Averaging intergroup/intragroup pairwise distances (the same way as with BD), we found that the sub-communities of butyrate producers in the group of 0–1 year old infants possess significantly larger degree of similarity to each other than the respective sub-communities within the group of 1–3 years old children and between these groups. This observation likely indicates that the butyrate producing bacterial communities are more mature and stable in older children. In contrast, the SCFA producer sub-communities have similar PBD and rPBD values for the groups of healthy and T1D samples, suggesting that the SCFA phenotypes cannot serve as biomarkers for the T1D status.

### 3.5 SCFA production capabilities and metabolomics data

**FIGURE 6:**
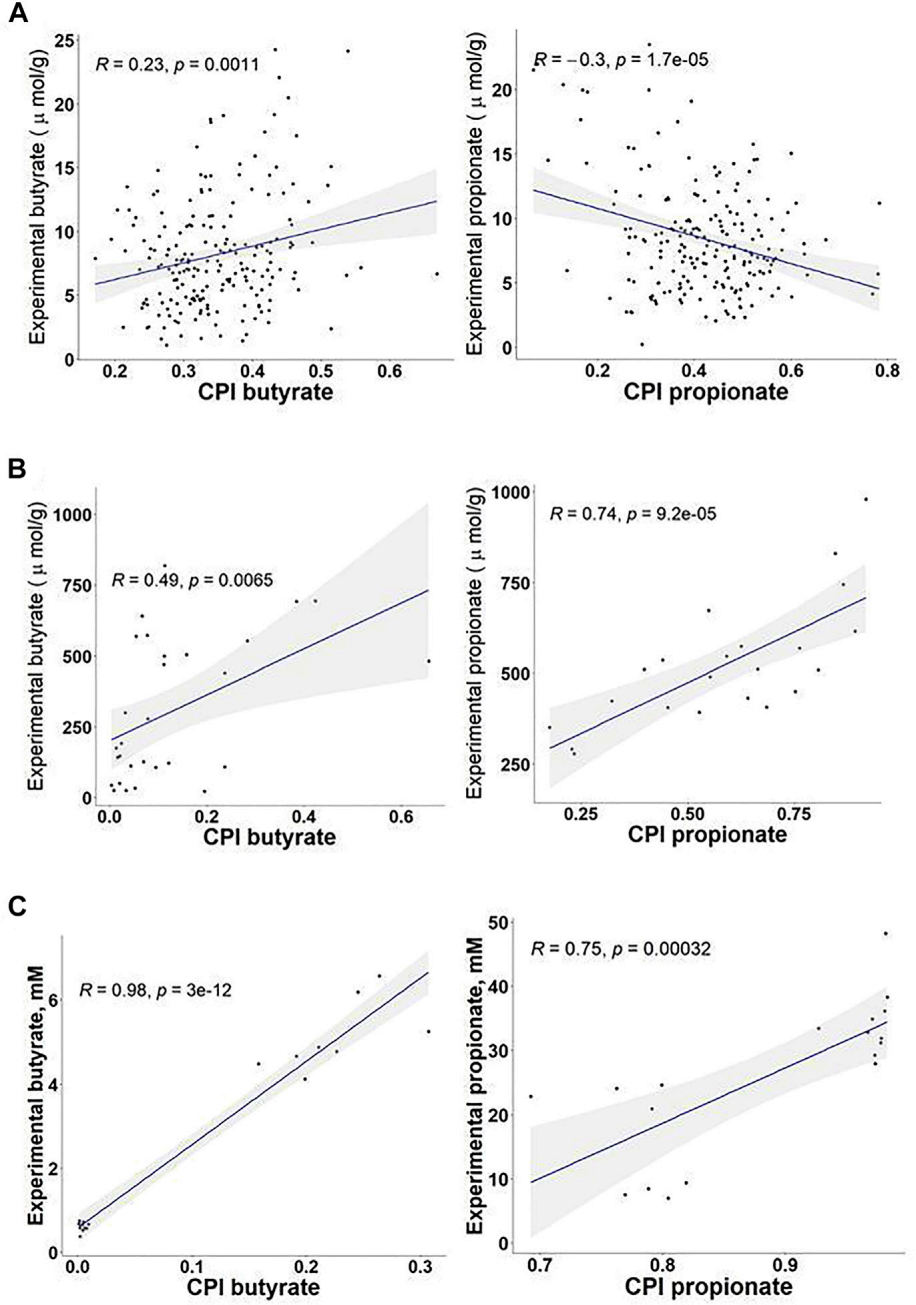
Correlations between Community Phenotype Indices (CPI) for butyrate and propionate production and the experimentally measured concentrations of SCFAs in 16S metagenomics studies of HGM. **(A)** *In vivo* study of the effects of dietary fibers on fecal microbiota of 200 healthy individuals (Deehan et al., 2020). **(B)** *In vitro* batch fermentation study of the effect of fibers on HGM microbiota (Chen M. et al., 2020). **(C)** Study of the effects of dietary emulsifiers on fecal microbiota *in vitro* (Elmén et al., 2020).

To understand relationship between the predicted SCFA production potentials and measured metabolic product concentrations we analyzed published microbiome datasets with available 16S metagenomics and metabolomics data. These include an *in vivo* study of HGM modulation with discrete dietary fiber structures in randomized controlled trial in humans (Deehan et al., 2020), and two *in vitro* fermentation studies describing the effect of dietary fibers and emulsifiers on bacteria in human fecal samples (Chen M. et al., 2020; Elmén et al., 2020). In each of these studies, we focused on the experimentally measured concentrations of butyrate and propionate and compared those with the corresponding CPI values calculated for 16S rRNA gene profiles using the Phenotype Profiler tool (Supplementary Table S4). Acetate was excluded from this analysis as the most abundant SCFA produced by a large number of HGM species. In the first *in vivo* study (Deehan et al., 2020), the experimentally measured concentrations of butyrate and propionate in fecal samples did not show significant Spearman correlation with the calculated CPI values of the fecal microbiota, suggesting the efficient absorption of SCFAs in the colon may dramatically reduce their fecal concentrations (Figure 6A).

On the contrary, CPI values correlate well with the measured SCFA concentrations obtained in experiments on *in vitro* fermentation of fecal inoculum. In the first *in vitro* study, the authors have performed batch fermentations of feces from 30 individuals on fiber substrates with different degrees of polymerization, namely carboxymethylcellulose, β-glucans, and galactooligosaccharides (Chen M. et al., 2020). The Spearman correlation coefficients between the experimentally measured concentrations and the calculated CPI values were 0.74 and 0.49 for propionate and butyrate, respectively (Figure 6B). In another *in vitro* study, fecal samples from twelve healthy human subjects have been fermented in a rich medium supplemented with or without sodium stearoyl lactylate (SSL) (Elmén et al., 2020). Measurement of SCFA concentrations has revealed that the SSL emulsifier reduces 6-8 fold the capacity of the bacterial inoculum to produce butyrate, while the production of propionate was increased 3-4 fold. The corresponding SSL-exposed microbiomes showed a significant increase in the relative abundance of propionate producers and dramatic reduction of butyrate producers. Comparison between corresponding CPI values and measured SCFA concentrations in this *in vitro* study showed the Spearman correlation coefficients 0.75 and 0.98 for propionate and butyrate, respectively (Figure 6C), providing a strong validation of the genomic-based approach to predict butyrate and propionate producers in HGM.

### 3.6 Comparison between the predictive metabolic profiling and pathway abundance approaches

**FIGURE 7:**
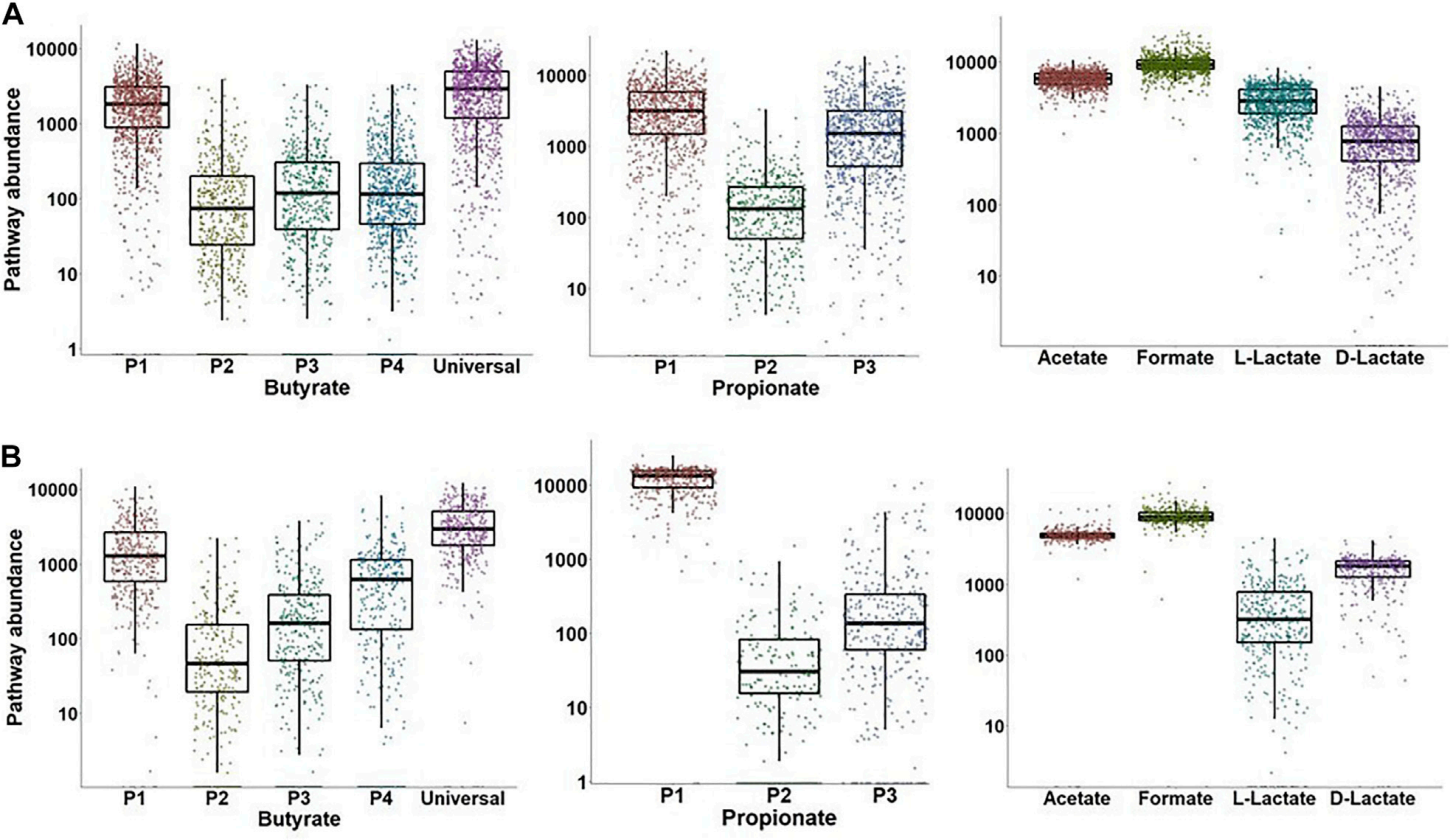
Distribution of metagenomic abundances for SCFA synthesis pathways in HGM samples from TEDDY **(A)** and IBD **(B)** datasets. Pathway abundances were calculated as a sum of TMM-normalized counts for selected signature genes in each SCFA pathway (see Supplementary Table S6).

To compare the distribution of metabolic genes from individual SCFA production pathways we analyzed two shotgun metagenomic sequencing datasets in WGS format, namely 1,048 samples from the German cohort of TEDDY dataset (Stewart et al., 2018) and 384 samples from the IBD study (Morgan et al., 2012). Pathway abundances were estimated as a sum of TMM-normalized gene abundances for functional roles that are specific to the P1, P2, P3, and P4 butyrate pathways and the P1, P2, and P3 propionate pathways (Supplementary Table S6). In addition, we calculated the TMM-normalized cumulative gene abundances for the universal butyrate pathway that converts Crotonyl-CoA to butyrate (Figure 1), as well as for the acetate, formate, L-lactate and D-lactate fermentation pathways. As result, the P1 (acetyl-CoA) and universal butyrate pathways are highly abundant in both IBD and TEDDY datasets, while the P2 (succinate), P3 (glutamate), and P4 (lysine) pathways are substantially underrepresented (Figure 7). Interestingly, the median value of the P4 (lysine) pathway is greater in the IBD dataset than in the TEDDY dataset, suggesting that adults have more producers synthesizing butyrate *via* the amino acid pathway than children. The P1 (succinate) pathway is the most abundant propionate synthesis pathway in both datasets, followed by the P3 (propanediol) pathway in children from the TEDDY dataset (Figure 7). In contrast, the P2 (lactate) pathway abundances are very low in both datasets, as well as the P3 pathway in adults from the IBD dataset. The P1 to P3 mean pathway abundance ratio is much higher in the IBD dataset, suggesting that children microbiomes contain the comparable numbers of producers synthesizing propionate from succinate and propanediol, while the P1 (succinate) pathway is predominant in adult gut microbiomes. Finally, mean abundances of acetate, formate, and D-lactate pathways are similar in both datasets, while the L-lactate pathway mean abundance is higher in the TEDDY dataset (Figure 7). We further obtained taxonomic profiles for each sample from the TEDDY and IBD datasets and mapped the obtained taxonomies to the reference HGM genomes in order to calculate the specific CPI values for each SCFA production phenotype (Supplementary Table S6). Comparison of the obtained CPI values with the TMM-normalized SCFA pathway abundances revealed a strong correlation with total abundance of genes from most abundant SCFA pathways, namely the P1 (acetyl-CoA) pathway for butyrate production, the P1 (succinate) pathway for propionate synthesis, as well as for L- and D-lactate synthesis pathways in both datasets (Table 3).

**TABLE 3:** Correlation coefficients between Community Phenotype Indices (CPI) and SCFA pathway abundances in the TEDDY and IBD WGS datasets.

SCFA	Pathway variant	Correlation coefficient	
		TEDDY	IBD
Butyrate	P1	0.69	0.73
	P2	0.00	0.16
	P3	0.18	0.31
	P4	0.13	0.11
	Universal	0.89	0.88
Propionate	P1	0.70	0.84
	P2	0.15	−0.11
	P3	0.47	−0.16
Acetate		0.37	0.26
Formate		0.16	0.24
L-lactate		0.76	0.84
D-lactate		0.83	0.77

To compare the phenotype profiling using cumulative CPI values with alternative approaches for prediction of metagenomic functions in 16S datasets, we chose the PICRUST2 algorithm allowing reconstruction of an ancestral state for gene occurrences or functional traits (Douglas et al., 2020). First, we used the obtained in this work BPM describing SCFA production phenotypes as a custom input of functional traits in PICRUST2. As result, we calculated the predicted SCFA phenotype abundances in 16S samples from the AGP and UKT datasets and compared them with corresponding CPI values. A fairly high Spearman correlation coefficients were obtained for butyrate and propionate in AGP dataset (0.5 and 0.61) and also in UKT dataset (0.56 and 0.81, respectively). We then compared the PICRUST2-predicted phenotype profiles with relative abundances of corresponding SCFA production metabolic pathways in MetaCyc obtained *via* the default PICRUST2 pipeline (Supplementary Table S7). We have selected metabolic pathways in the MetaCyc database that most accurately describe of butyrate and propionate biosynthesis capabilities. Formate, acetate and lactate pathways were not included in this comparison, since the corresponding enzymes participate in multiple central pathways. The best Spearman correlation coefficients were observed for the P1 (acetyl-CoA) butyrate pathway and the P1 (succinate) propionate pathway in HGM samples from both the AGP and UKT datasets (Table 4).

**TABLE 4:** Correlation coefficients between phenotype abundances produced by the PICRUSt2 pipeline with binary phenotypes and MetaCyc pathway abundancess in AGP and UKT datasets.

SCFA	Pathway variant	BioCyc ID	Correlation		MetaCyc pathway description
			AGP	UKT	
Butyrate	P1	PWY-5676	0.73	0.43	acetyl-CoA fermentation to butanoate II
	P2	PWY-5677	0.45	−0.05	succinate fermentation to butanoate
	P3	P162-PWY	0.5	0.05	L-glutamate degradation V *via* hydroxyglutarate
	P4	P163-PWY	0.36	−0.01	L-lysine fermentation to acetate and butanoate
Propionate^a^	P1	P108-PWY	0.93	0.92	pyruvate fermentation to propanoate I
	P3	PWY-7013	0.37	0.15	(S)-propane-1,2-diol degradation

a Propionate pathway variant P2 (acrylate pathway, PWY-5494) was not present in the PICRUST2 output.

## 4 Discussion

The human gut microbiota produces a large number of metabolites that influence our health and physiology. SCFAs are produced in the large intestine as fermentation products from dietary fibers and proteins. Acetate, propionate and butyrate are the most abundant products, representing 90–95% of the microbially produced SCFAs. Other fermentation products, including formate, succinate and two enantiomers of lactate, are produced by many HGM species but do not accumulate to high levels in the colon due to bacterial cross-feeding allowing their further conversion to acetate, propionate and butyrate. Intrinsic variability of taxonomic composition of HGM that is caused by many variable environmental factors such as diet, lifestyle and drug use, translates to extensive variations of HGM functional potentials. Genomic-based prediction of HGM metabolic potential (such as SCFA production) based on 16S or WGS metagenomic data is important for microbiome research and development of personalized prebiotics and probiotics. Isolating and culturing of individual HGM bacterial species to identify their metabolic products is not feasible due to unknown carbon sources and growth medium conditions for a significant number of HGM species. Thus, development of bioinformatics approaches for prediction of SCFA production potential is important for predictive functional profiling of microbiomes. In our previous works, we developed the metabolic phenotype profiling approach to estimate the relative abundance of HGM bacteria encoding functional variants of amino acid and vitamin biosynthetic pathways (Rodionov et al., 2019; Ashniev et al., 2022), while here we expanded it for predictive profiling of SCFA production capabilities in HGM metagenomes.

To obtain the reference collection of SCFA metabolic genes and phenotypes we utilized the subsystems approach and analyzed a set of 2856 HGM genomes representing 823 bacterial species. As result, the comparative genomics analysis allowed us to reconstruct all previously described variants of metabolic pathways for production of butyrate (4 variants), propionate (3 variants), formate, acetate, and lactate (Figure 1). Complete pathways of butyrate and propionate synthesis were identified in 359 and 826 genomes, respectively, at that 167 and 122 of them contain more than one pathway variant for each SCFA, respectively (Table 1). These include five alternative enzymes from propionate synthesis pathways represented by non-orthologous gene displacements. Each of the formate, acetate and lactate production pathways contains one or two enzymes and are much broader distributed among the analyzed HGM genomes (45–87%). To validate the predicted SCFA phenotypes, we collected published experimental data on SCFA production capabilities in 210 HGM species, and obtained 96 and 85% consistency between experimental and predicted phenotypes for butyrate and propionate, respectively (Supplementary Table S2). We also compared our reference HGM collection of butyrate producers with the results of previous bioinformatics studies. Vital et al. established a gene catalogue of butyrate-producing pathways by screening 3,184 sequenced bacterial genomes from the Integrated Microbial Genome database by specific EC numbers from KEGG database and by using Hidden Markov Models (HMM) models for corresponding protein families (Vital et al., 2014). As result, the authors identified 225 bacterial genomes of butyrate producers, 107 of which are environmental isolates and the remaining 117 out of 118 genomes are also present in our reference collection of butyrate producers (Supplementary Table S1). Overall, our expanded collection of HGM genomes contains 242 additional predicted butyrate producers as compared to the list of butyrate producers identified by Vital et al.

The obtained reference collection of SCFA production pathways and genes was further used for functional profiling of HGM metagenomes from published datasets. This analysis included the calculation of sample-specific phenotype abundance CPI values (for 16S and WGS datasets) and overall metabolic pathway abundance (for WGS). Each CPI value represents a probabilistic estimate of the fraction microbial cells that are capable to produce a specific SCFA. To enable the phenotype profiling approach, we obtained the simplified binary phenotypes corresponding to producers (“1”) and non-producers (“0”) of each SCFA. These binary values were combined together for 2,856 analyzed genomes to make a BPM that was further analyzed across taxonomically-related genomes to determine the variability of SCFA phenotypes at the species and genus levels (Supplementary Table S3). Comparison of binary SCFA phenotypes across 823 HGM species and 296 genera revealed that ∼8% of species and 35% of genera have at least one variable phenotype and that majority of them belong to the Firmicutes phylum (Table 2). Most of these variations can be explained by either strain-specific gain or loss of SCFA production pathway genes that are often organized into operons and are frequent subject to a horizontal transfer. For example, the P3 (propanediol) pathway for propionate production encoded by the *pdu* gene cluster was identified only in 9% of 144 analyzed *Lactobacillus* genomes, at that this pathway is present in 2 out of 3 strains of *L. brevis* and 8 out of 10 strains of *L. reuteri*. To minimize the CPI prediction error due to the observed phenotype variations we used the sequence-based mapping scheme for functional profiling of 16S microbiome datasets. As result, mean CPI prediction error values were no more than 1% for most analyzed 16S datasets and SCFA phenotypes (Supplementary Table S4).

Analysis of the AGP and UKT datasets showed significant similarity between the obtained SCFA phenotype profiles (Figure 2), which may be related to similar dietary patterns (consumption of fibers, proteins, vitamins) in these two Western populations. Both datasets show high average CPI values for acetate and formate and low values for butyrate and L-lactate. Unlike the European and American microbiomes, gut microbiomes of the indigenous hunter-gatherers from Tanzania have reduced CPI values for butyrate, propionate, and lactate production. Overall, our observations suggest that the potential for the production of terminal SCFAs in the Hadza gut communities is shifted towards acetate and formate, with the possibility of their conversion into other SCFAs through the process of cross-feeding (see below). At the same time, we conclude that the landscape of probable fermentation products is more diverse in the AGP and UKT gut communities, with presumably more intricate cross-feeding networks. The comparison of CPI profiles for two age groups of young children from the Sweden cohort of the TEDDY dataset revealed an increased number of butyrate producers in the group of older children (1–3 years old) compared to younger infants (Figure 4), indicating a stabilization of the butyrate production phenotype in the older children population. The observed large shifts in the predicted SCFA production profiles during infant gut microbiota development agree well with the previous measurements of fecal major fermentation products and microbial families that were linked to the introduction of complementary foods around the age of 6 months followed by reduction and eventual cessation of breastfeeding in infants after 1 year of age (Vatanen et al., 2018; Appert et al., 2020). Relative abundance of top butyrate-producing species demonstrated gradual increase with the children age in the TEDDY cohort (Figure 5). Similar trends of increasing abundance of major butyrate producers and fecal butyrate levels were recently reported for a cohort of 3–12 months old children from Switzerland (Appert et al., 2020). We further applied a diversity-based approach for the detection of SCFA phenotypes that are associated with children’s age and diagnosis. By comparing relative PBD metrics for each SCFA phenotype, we found that sub-communities of butyrate producers in the 0–1 year old group possess a significantly larger degree of similarity to each other than the respective sub-communities within the 1–3 years old group and between these groups. It is noteworthy that similar observation was made in our previous work (Iablokov S. et al., 2021), where we established that PBD for butyrate was lower in “healthy” (vs. “Crohn’s disease”) group, thus making the respective sub-communities of butyrate producers follow the famous “Anna Karenina” principle for microbiomes (Ma and Sam, 2020). If applied to the present analysis, this principle could be restated for butyrate as follows: as the child gut microbiome matures, the microbial communities become more similar to one another, however, only in terms of butyrate-producing species.

To validate the functional profiling approach, we analyzed three published HGM datasets with measured SCFA concentration data for each 16S metagenomic samples. Quantitative assessment of SCFAs in fecal samples *via* metabolomics reflects steady state metabolite levels, however 90–95% of SCFAs produced in the colon lumen are absorbed by the intestinal mucosa (McNeil et al., 1978). Indeed, our analysis of published metabolomic and metagenomic data showed a correlation between CPI values and concentrations for butyrate and propionate measured in *vitro* fermentation experiments (Figure 6). However, the HGM datasets with *in vivo* measured SCFA concentrations in feces did not show a correlation with corresponding CPI values. Finally, we compared the predictive metabolic profiling with pathway abundance approaches by analyzing two WGS datasets with our expanded collection of SCFA synthesis genes from 2,856 reference HGM genomes. The calculated TMM-normalized gene abundances were summed up for each individual SCFA pathway (Figure 7) and compared to the CPI profile, resulting in significant correlation coefficients for butyrate (P1), propionate (P1), L-, and D-lactate pathways (Table 3). In a previous work, Vital et al. also used the SCFA production pathway gene catalogue for functional and taxonomic profiling of butyrate producers in HGM samples from 15 metagenomic datasets (Vital et al., 2017). The P1 (acetyl-CoA) pathway genes were most abundant in samples derived from healthy individuals of nine metagenomics studies, and *F. prausnitzii* was the major species contributing to this pathway abundance. These previous results are in agreement with our current results on the abundance of individual butyrate pathways in the TEDDY and IBD datasets.

We also compared our SCFA phenotype profiling approach with a state-of-the-art functional profiling approach for 16S metagenomics datasets, PICRUSt2, which uses an ancestral-state reconstruction algorithm and the MetaCyc pathway’s collection (Douglas et al., 2020). The PICRUSt2-predicted relative abundances of the major butyrate (P1 or acetyl-CoA) and propionate (P1, succinate) production pathways revealed a generally good agreement with the corresponding CPI values in both AGP and UKT datasets (Table 4). However, the abundances of alternative butyrate and propionate production pathways did not correlate with the predicted community phenotype values. The latter results can be explained by significantly lower abundances of the P2/P3/P4 pathways for butyrate and P2/P3 pathways for propionate in HGM samples, which is supported by our quantitative analysis of two WGS datasets (Figure 7) and by the previous analysis of 15 WGS datasets for butyrate (Vital et al., 2017).

**FIGURE 8:**
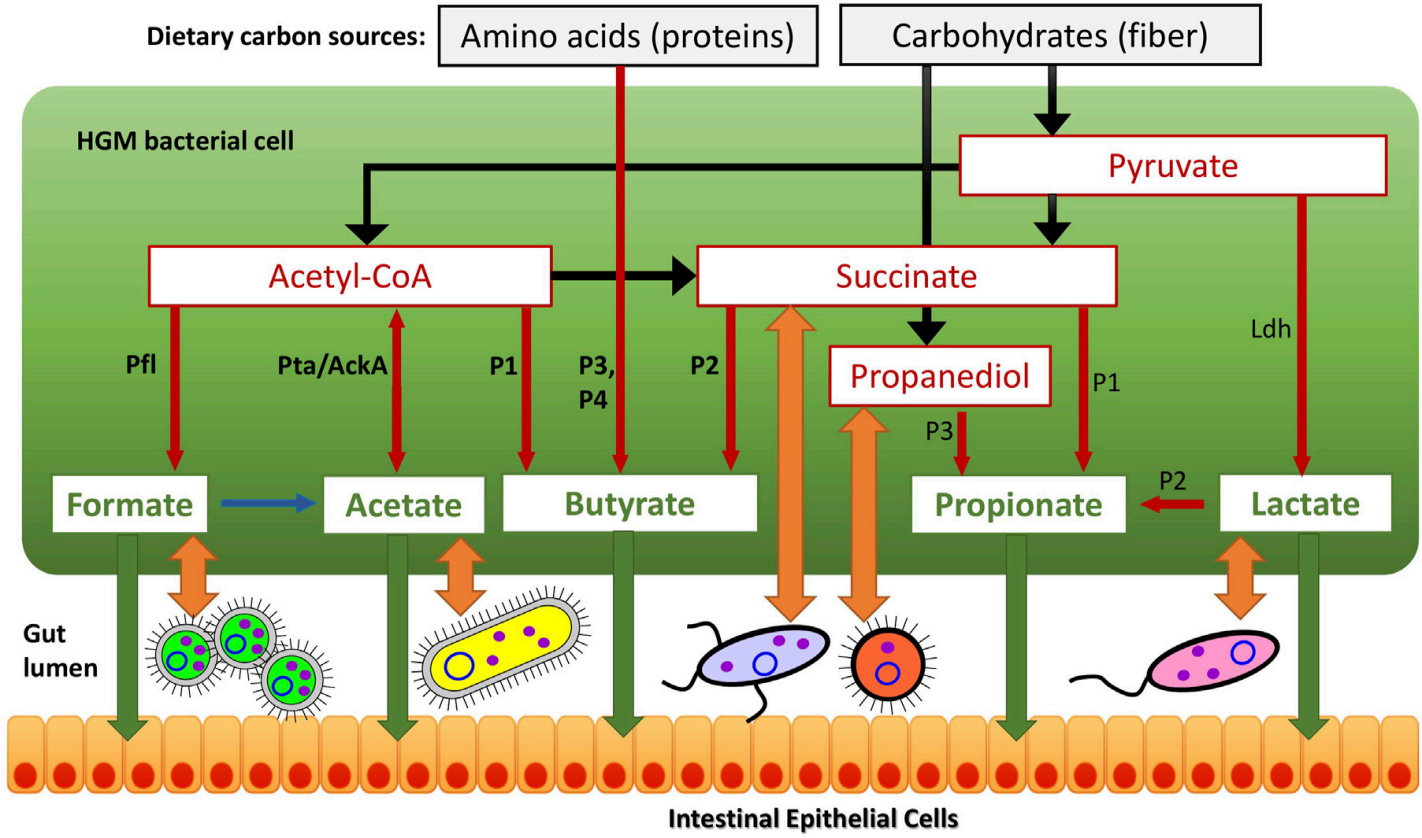
Metabolic pathways and cross-feeding mechanisms for SCFA production by HGM bacteria. Terminal SCFAs and lactate are in green. Dietary nutrients and core metabolic precursors are in black and red, respectively. Microbial SCFA fermentation pathways analyzed in this work are shown by red arrows. Carbohydrate catabolic pathways are in black. Wood-Ljungdahl pathway is in blue. Absorption of terminal SCFAs by intestinal epithelial cells is shown by thick green arrows. Cross-feeding interactions between HGM members are shown by thick orange arrows.

One limitation of our phenotype profiling approach is that it does not take into account metabolite cross-feeding interactions in HGM communities, when certain microorganisms are capable to consume SCFAs or their metabolic precursors produced and secreted by other community members. Figure 8 summarizes all known metabolic interactions between HGM species contributing to accumulation or usage of SCFAs/precursors. First, lactate and acetate produced from carbohydrates by diverse HGM species (such as *Bifidobacterium* and *Lactobacillus* spp.) serve as substrates for formation of propionate (*via* P2 pathway) and butyrate (through acetyl-CoA, and P1 pathway) by numerous other HGM species. These metabolic interactions were confirmed in a few *in vitro* co-culturing studies, including 1) the co-growth of *B. adolescentis*, which uses starch and fructose oligosaccharides as substrates, and strains of *Eubacterium (Anaerobutyricum) hallii* and *Anaerostipes caccae*, that use lactate and acetate (formed by *B. adolescentis*) and produce butyrate as a final SCFA product (Belenguer et al., 2006); and 2) the co-culture of acetate-producing *B. longum* and *Roseburia intestinalis* that is capable to produce butyrate from exogenous acetate (Falony et al., 2006). The second important intermediate for cross-feeding in HGM communities is succinate, which is a common product of bacterial fermentation of dietary fiber. The main producers of succinate in the intestine are bacteria from the Bacteroidetes phylum, mainly *Bacteroides* and *Prevotella* (De Vadder et al., 2016; Connors et al., 2018). Succinate is usually found at relatively low concentrations in the intestinal lumen, since it is a major substrate for the formation of propionate and an additional metabolic precursor for butyrate (Louis and Flint, 2017). The conversion of succinate to propionate (*via* the P1 pathway) was previously mainly studied in several Firmicutes bacteria from the Negativicutes class (Reichardt et al., 2014), while the butyrate production from succinate (*via* the P2 pathway) was demonstrated in *Porphyromonas gingivalis* and *Clostridioides difficile* (Louis and Flint, 2017). The third strategy of cross-feeding relationships in HGM implies the propionate-producing strains possessing the P3 (propanediol) pathway (Table 1). 1,2-propanediol is a major end product from anaerobic degradation of L-rhamnose or L-fucose by various HGM species from the *Anaerostipes*, *Bacteroides*, *Clostridium*, and *Escherichia* genera (Baldomà and Aguilar, 1988; Rodionova et al., 2013). The other routes of synthesis of 1,2-propanediol are from other sugars *via* the glycolysis intermediate dihydroxyacetone-phosphate and methylglyoxal (Saxena et al., 2010). Several microorganisms, including *Salmonella typhimurium* (Staib and Fuchs, 2015) and *Roseburia inulinivorans* (Scott et al., 2006), are capable to directly catabolize fucose to propionate *via* 1,2-propandiole, while other HGM bacteria use 1,2-propandiole to form cross-feeding relationships with each other. For example, 1,2-propandiole produced by *Bifidobacterium breve* is utilized by *Lactobacillus reuteri* to convert it to propionate (Cheng et al., 2020). Finally, formate is produced by many HGM species and further converted to acetate *via* the Wood-Ljungdahl pathway in acetogenic clostridia; indeed co-cultivation on starch of the formate-producing *Ruminococcus bromii* and the acetogenic bacterium *Blautia hydrogenotrophica* led to the disappearance of formate and an increase quantity of acetate (Laverde Gomez et al., 2019).

Taking together, the reconstructed biochemical pathways and binary metabolic phenotypes for SCFA and lactate production in 2,856 reference HGM strains allowed us to expand the previously reported BPMs for amino acid and vitamin biosynthesis and to perform a quantitative analysis of SCFA phenotype profiles in HGM metagenomes. The predicted SCFA synthesis phenotype and pathway abundances can find its practical application in the diagnosis and treatment of syndromes associated with dysbiosis through a rational and personalized choice of probiotics and food supplements. Future expansion of the reference genome set by newly sequenced HGM isolates and metagenome-assembled genomes will improve the accuracy and reliability of our functional phenotype profiling approach.

## Data Availability

Publicly available datasets were analyzed in this study. This data can be found here: www.ebi.ac.uk/ena, project IDs PRJEB11419 (AGP), PRJEB13747 (UKT), PRJEB27517 (Hadza), PRJNA400115 (TEDDY), PRJNA398089 (IBD), PRJNA560950 (Deehan), PRJNA573754 (Chen), PRJNA600537 (Elmen).
