# Supplementary material for: Genomic reconstruction of short-chain fatty acid production by the human gut microbiota
Source: Front Mol Biosci. 2022 Aug 11;9:949563. doi: 10.3389/fmolb.2022.949563 (PMC9403272; doi:10.3389/fmolb.2022.949563)
Supplement: Supplementary file 3 [file Image2.PDF]

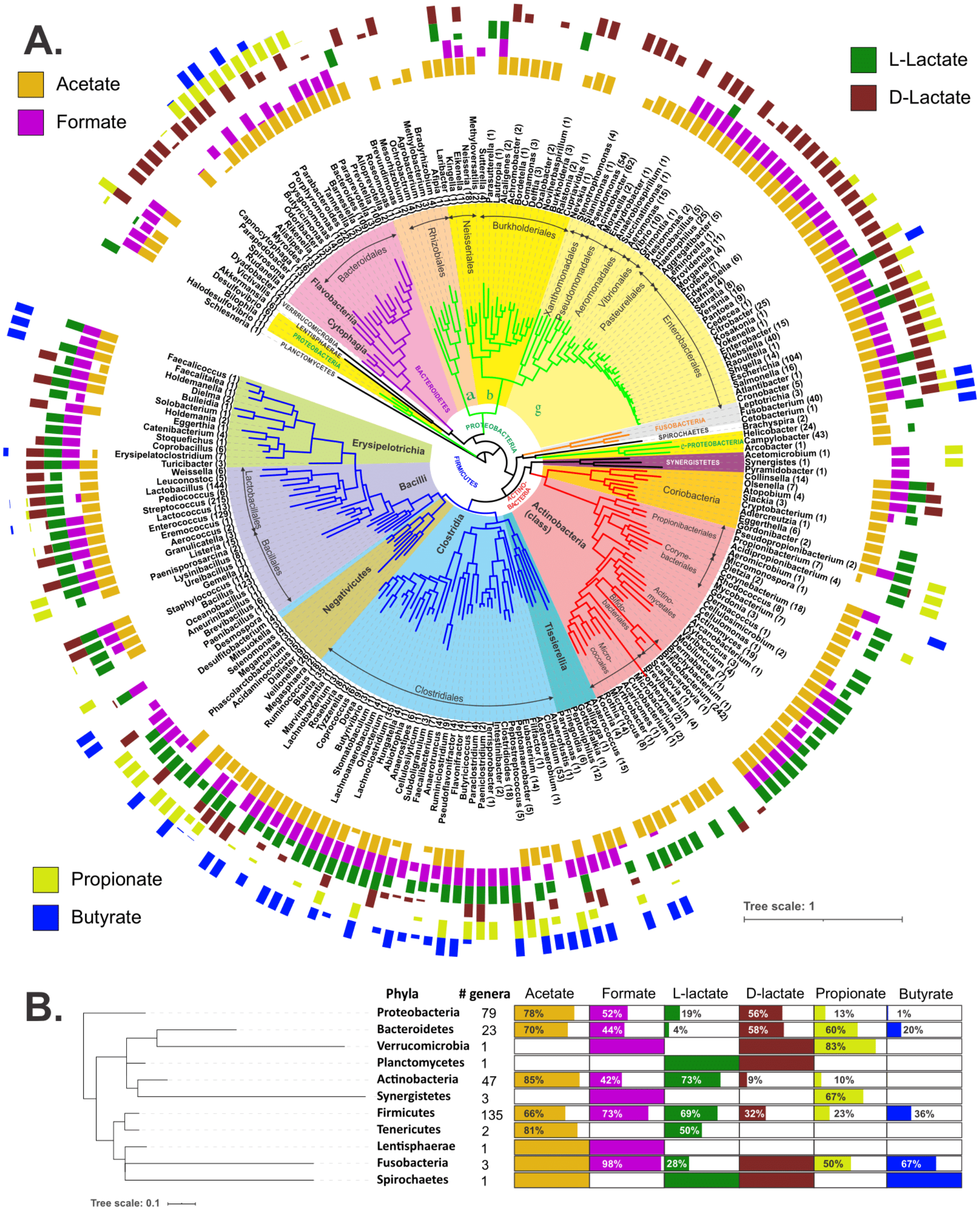

Supplementary Figure S2. **(A)** The distribution of SCFAs producers among analyzed HGM strains at the genus level. Colored bars show the average SCFA production phenotypes of each genus, empty bars represent non-producers. The phylogenetic tree of HGM genera was built using concatenated alignment of ribosomal proteins (see Methods). The number of analyzed genomes per genus is shown in parenthesis. **(B)** The distribution of SCFAs producers at the phylum level. The number of analyzed genera in each phylum is shown.
