# Supplementary material for: Genomic reconstruction of short-chain fatty acid production by the human gut microbiota
Source: Front Mol Biosci. 2022 Aug 11;9:949563. doi: 10.3389/fmolb.2022.949563 (PMC9403272; doi:10.3389/fmolb.2022.949563)
Supplement: Supplementary file 5 [file Image3.PDF]

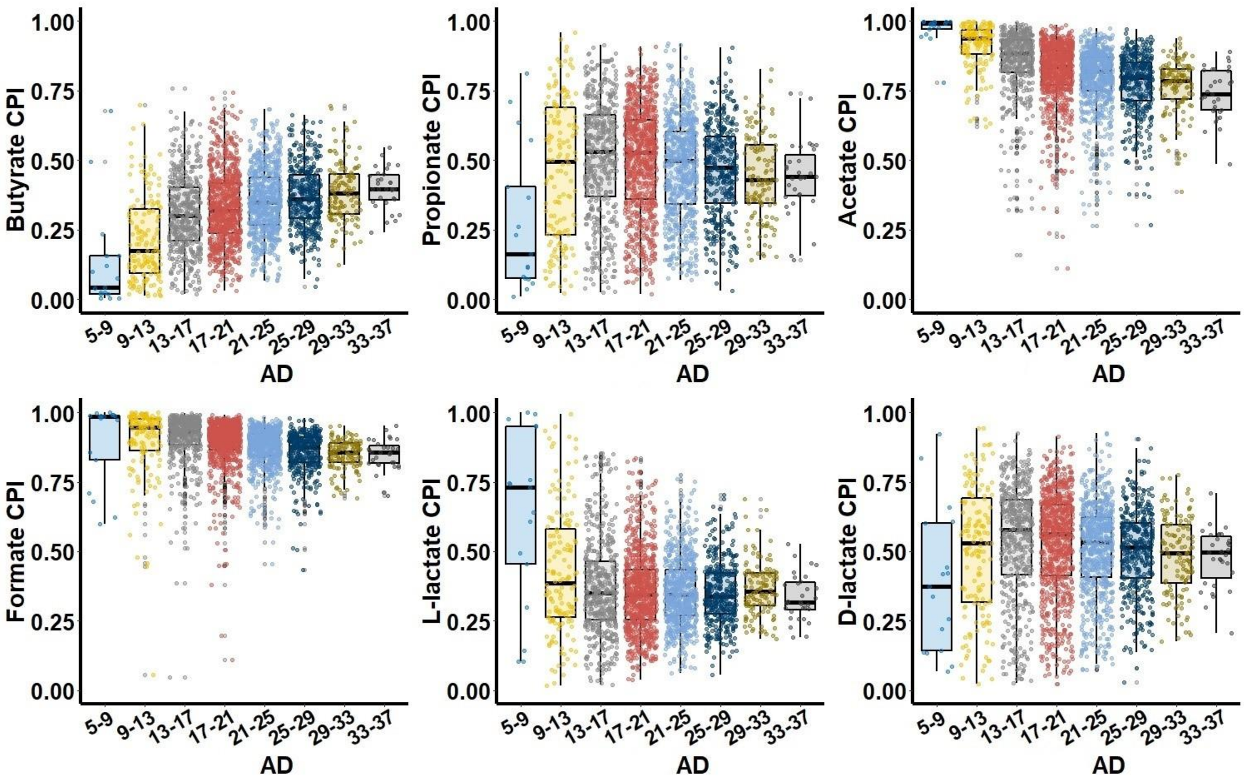

Supplementary Figure S3. Community Phenotype Indices (CPI) versus Alpha Diversity (AD) scatterplots for SCFAs and two forms lactate metabolic phenotypes calculated for the AGP dataset. Samples are grouped together based on their AD values calculated using Faith phylogenetic diversity metric.
