# Supplementary material for: Genomic reconstruction of short-chain fatty acid production by the human gut microbiota
Source: Front Mol Biosci. 2022 Aug 11;9:949563. doi: 10.3389/fmolb.2022.949563 (PMC9403272; doi:10.3389/fmolb.2022.949563)
Supplement: Supplementary file 10 [file Image1.PDF]

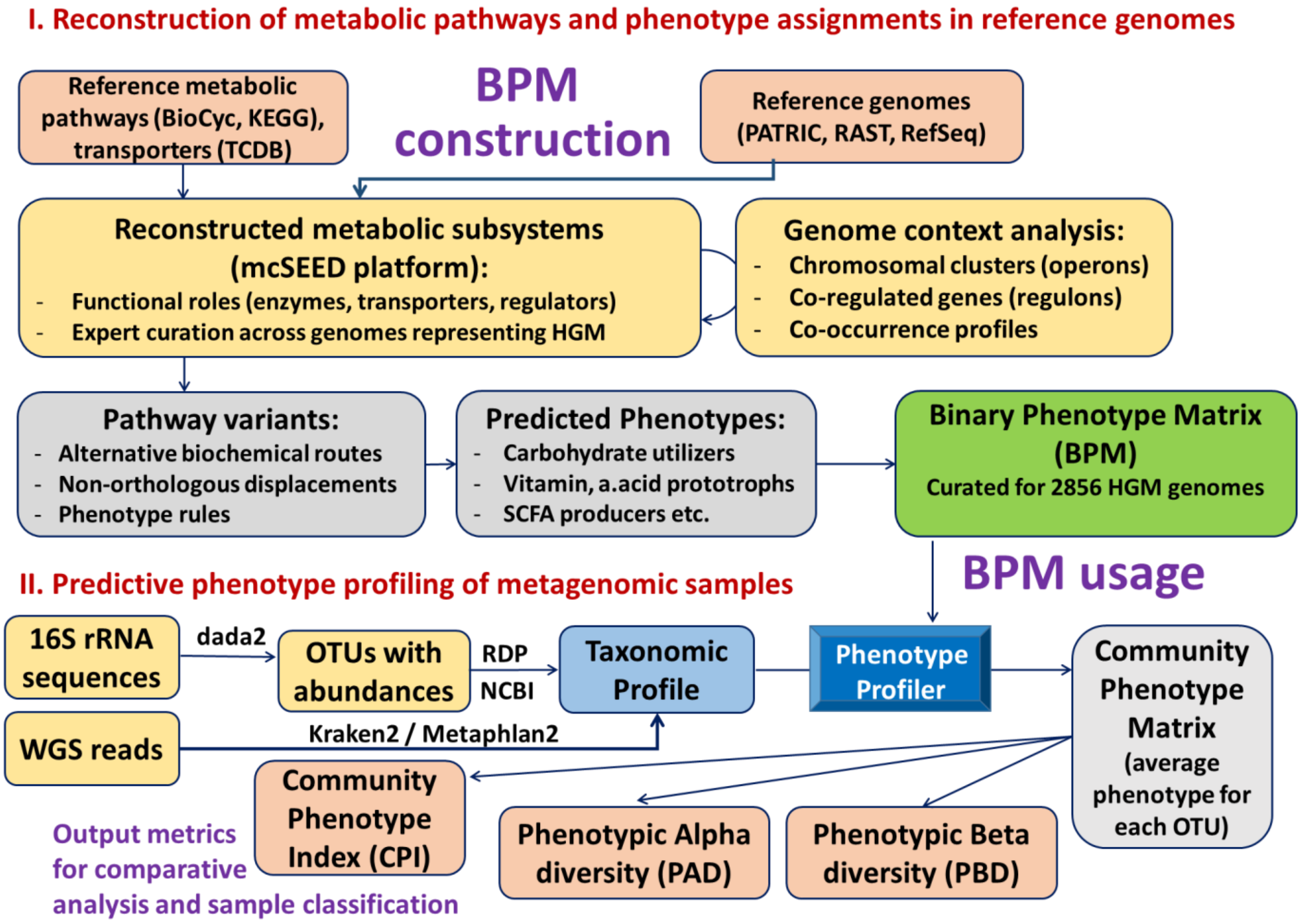

Supplementary Figure S1. The bioinformatics workflow for metabolic phenotype profiling of the Human Gut Microbiome (HGM). (I) Comparative-genomics based workflow used for recosntruction of SCFA synthesis metabolic pathways and construction of Binary Phenotype Matrix (BPM) in the reference set of HGM genomes. (II) Computational pipeline for predictive phenotype profiling of metagenomic samples (16S or WGS).
